# SUMOylation-Driven Subtype Heterogeneity and Prognostic Biomarkers in Renal Cell Carcinoma

**DOI:** 10.3390/cimb48080751

**Published:** 2026-07-23

**Authors:** Xiaobo Zhang, Zhiming Li, Ruoxin Lin, Suping Yang, Xiaohui Sun, Shicheng Chen

**Affiliations:** 1 Department of Bioengineering and Biotechnology, College of Chemical Engineering, Huaqiao University, Xiamen 361021, China; ghinazy@163.com (X.Z.); yangsuping@hqu.edu.cn (S.Y.); 2 Cuiying Biomedical Research Center, The Second Hospital & Clinical Medicine School, Lanzhou University, Lanzhou 730030, China; 3 State Key Laboratory of Genetics and Development of Complex Phenotypes, School of Life Sciences, Fudan University, Shanghai 200438, China; 4 Medical Laboratory Sciences Program, College of Health and Human Sciences, Northern Illinois University, DeKalb, IL 60115, USA

**Keywords:** renal cell carcinoma, SUMOylation, biomarkers, heterogeneity, prognostic

## Abstract

Renal cell carcinoma (RCC) is the most common malignancy of the urinary system, characterized by high incidence, mortality, and resistance to therapy. Its molecular heterogeneity presents challenges for effective precision treatment. RCC is highly heterogeneous, yet treatment guidelines rely predominantly on kidney renal clear cell carcinoma (KIRC) studies, neglecting other molecular subtypes, which limits therapy personalization for non-KIRC patients. This study aimed to explore the role of small ubiquitin-like modifier (SUMOylation)-associated genes in the progression and prognosis of RCC and its subtypes. We identified 298 SUMOylation-associated differentially expressed genes (DEGs), including 151 RCC-specific genes after excluding expression changes attributable to RCC subtype-specific variation. Ten core genes (*PRKCG*, *PRKCQ*, *PRKCD*, *IRS4*, *SLC2A4*, *SLC27A1*, *SLC27A4*, *LCN2*, *S100A7*, and *S100A9*) were identified, with *LCN2* expression not only discriminates tumor from normal tissue but also separates KIRC from KICH/KIRP, proposing *LCN2* as a potential second-step biomarker for KIRC identification on top of traditional histology. Inter-subtype RCC heterogeneity represented a key factor limiting predictive performance of the six prognostic signature genes (*CREB3L1*, *GNA11*, *PFKM*, *PPARGC1A*, *PPP2R2C* and *PRKCG*). Its 5-year AUC exceeded 0.7 for every individual RCC subtype in the TCGA training cohort, with pooled 5-year AUCs of 0.61 (TCGA training cohort) and 0.67 (independent PCAWG validation cohort). The prognostic risk model demonstrated strong predictive performance, with a C-index of 0.791 before calibration and 0.774 after calibration. Importantly, the C-index remained above 0.75 throughout the 60-month follow-up period, indicating stable and robust long-term prognostic accuracy. High-risk patients exhibited greater immune cell infiltration, indicating potential for immunotherapy. Following secondary screening, three RCC cell lines (BFTC909, CAKI1, and CAL54) and five target genes (*PRKCD*, *SLC27A1*, *SLC27A4*, *LCN2* and *GNA11*) were identified as optimal candidates for subsequent mechanistic investigations. This study uncovers the prognostic and functional relevance of SUMOylation in RCC and offers a novel framework for biomarker development, therapeutic targeting, and immunotherapeutic stratification.

## 1. Introduction

Renal cell carcinoma (RCC) is the most prevalent malignant tumor of the urinary system, comprising 80–90% of all renal malignancies [1]. Globally, its incidence and mortality rates are high, resulting in a significant disease burden [1,2]. RCC varies markedly across age and sex, with peak incidence occurring in individuals aged 60–70 years. Notably, the incidence in males is 1.5 times higher than in females [3]. Established risk factors include smoking, obesity, hypertension, chronic kidney disease, and environmental exposure to heavy metals and industrial solvents [4,5,6]. Furthermore, genetic factors contribute significantly to RCC pathogenesis, with several hereditary cancer syndromes strongly linked to RCC, including Von Hippel-Lindau (VHL) syndrome and Birt-Hogg-Dubé syndrome [7,8].

On the basis of its pathological characteristics, RCC can be classified into three major subtypes: kidney renal clear cell carcinoma (KIRC), kidney renal papillary cell carcinoma (KIRP), and kidney chromophobe renal cell carcinoma (KICH) [9]. KIRC is the most prevalent subtype, representing 75–80% of RCC cases, followed by KIRP (10–15%) and KICH (5%) [9]. KIRC derives its distinctive histopathological feature-abundant clear or pale cytoplasm due to cytoplasmic lipid and glycogen accumulation. With progress in molecular mechanism research, the latest World Health Organization (WHO) classification now includes over 20 malignant RCC subtypes. Traditional morphological methods alone are insufficient for accurate classification—for instance, while KIRP was initially classified morphologically into type 1 and type 2, molecular analyses have since revealed more precise subtypes, including distinct KIRP molecular subtypes and other special subtypes [10]. Although targeted therapy and immunotherapy have improved outcomes for some patients with advanced RCC, 20–40% respond poorly due to the disease’s pathological complexity and heterogeneity [1,11,12]. RCC is highly heterogeneous, yet treatment guidelines rely predominantly on KIRC studies, neglecting other molecular subtypes, which limits therapy personalization for non-KIRC patients [12,13]. A comprehensive understanding of RCC heterogeneity—including molecular subtyping, biomarker identification, and microenvironmental interactions—is essential for developing genotype-directed therapies and optimized combination strategies for personalized treatment [10,13].

In the pathogenesis of KIRC, the VHL tumor suppressor gene is inactivated in approximately 90% of cases [9,14]. The *VHL* gene, which maps to chromosome 3p, encodes a critical component of the E3 ubiquitin ligase complex that targets hypoxia-inducible factor (HIF) family subunits (HIF1α, HIF2α, and HIF3α) for proteasomal degradation [15]. VHL protein inactivation is primarily by deleterious mutations or partial deletion of chromosome 3p [16], leading to constitutive activation of hypoxia and angiogenesis-related pathways [17]. Consequently, KIRC tumors exhibit a hypervascular phenotype with an increased risk of hemorrhage [1]. The oxygen-dependent interaction between the VHL protein and HIFα constitutes the central regulatory mechanism for cellular hypoxia sensing and response (VHL-HIF pathway) [17]. Dysregulation of this pathway is a hallmark feature of RCC development and progression [1,17]. Building upon this regulatory framework, emerging evidence further suggests a functional crosstalk between the VHL-HIF pathway and protein SUMOylation, wherein SUMOylation enhances HIF stability by attenuating its VHL-mediated degradation. This interplay offers a mechanistic rationale for targeting SUMOylation to mitigate RCC subtype heterogeneity and advance therapeutic strategies [18].

To date, over 6000 potential SUMOylated substrates predominantly localized in the nucleus have been identified [17]. SUMOylation orchestrates essential cellular processes, including apoptosis, autophagy, and senescence, and contributes to the pathogenesis of various diseases. During apoptosis, SUMOylation modulates execution through precise regulation of apoptotic effector proteins [19]. SUMO1 deficiency in mice results in premature lethality and cardiovascular pathologies [20]. Importantly, SUMOylation form of Drp1 is isoform specific, where SUMO1–SUMO2/3 conjugation drives distinct functional consequences during ischemia-induced apoptosis [20]. In cancer biology, SUMOylation predominantly exerts antiapoptotic effects. Dysregulation of SUMOylation has been linked to the development of acquired multidrug resistance, highlighting the potential of restoring SUMOylation homeostasis as a novel therapeutic strategy in oncology [21]. As a critical stress-responsive mechanism, autophagy is tightly modulated through SUMOylation-mediated regulation [22]. The autophagy-initiating factor PIK3C3 physically associates with SUMO-conjugated PDPK1 and BECN1 to orchestrate autophagosome biogenesis [23]. SUMOylation regulates senescence by posttranslationally modifying the key regulator promyelocytic leukemia nuclear bodies (PML-NBs), promoting their structural integrity and functional activation, such as through modification at lysine 160 (K160). This process also facilitates broader senescence programs, including p53 SUMOylation and the establishment of reinforcing feedback loops [24,25,26]. Given its central role in both tumor biology and aging, pharmacological modulation of the SUMOylation pathway presents a promising avenue for therapeutic intervention in cancer and age-related diseases [20].

In this study, we conducted a comprehensive molecular profiling analysis of RCC, with a particular focus on alterations in SUMOylation-associated gene signatures. We further characterized the inter-subtype heterogeneity among the three major RCC variants and developed a robust prognostic risk model. In addition, we systematically examined the tumor immune microenvironment (TIME) landscape across these distinct RCC subtypes and identified potential cell lines and target genes for further in-depth molecular biological investigations. Collectively, our findings offer novel therapeutic insights and support precision medicine strategies tailored to specific RCC subtypes.

## 2. Materials and Methods

### 2.1. Data Source

An initial set of 4817 SUMOylation-associated genes was retrieved from the GeneCards database (https://www.genecards.org/ (accessed on 20 June 2025)). To minimize selection bias, no predefined GeneCards relevance score cutoff was applied during the initial gene collection. Instead, the complete gene set was retained for unbiased downstream screening, thereby avoiding the premature exclusion of moderately annotated genes that may play important roles in renal cell carcinoma (RCC). Subsequently, a stringent multi-step filtering strategy integrating transcriptional, functional, and clinical evidence was implemented to eliminate genes with weak or incidental associations with SUMOylation. Candidate genes were required to satisfy the following five criteria to ensure biological relevance and interpretability: (1) annotation in SUMOylation-related databases; (2) significant differential expression between RCC and normal kidney tissues; (3) robust diagnostic performance for distinguishing tumor from normal tissues across all three major RCC subtypes (KICH, KIRC and KIRP); (4) enrichment in SUMOylation-related biological processes or pathways, indicating strong functional relevance to SUMOylation; and (5) evidence of protein–protein interactions with SUMOylation-associated proteins and/or demonstrated clinical prognostic significance.

Based on this list of genes, gene expression profiles and clinical data for 891 RCC patients and 129 normal individuals were extracted from The Cancer Genome Atlas (TCGA) database (https://portal.gdc.cancer.gov (accessed on 25 June 2025)). Transcriptomic data were merged with corresponding clinical records using patient identifiers, and 133 cases were excluded due to duplication or mismatched information. This curation resulted in a final dataset comprising complete gene expression and clinical data for 758 RCC patients and 129 normal controls within the TCGA cohort. The clinical data encompassed key variables, including sex, age, survival status, and overall survival (OS).

Construction of the prognostic risk model was performed using a TCGA training cohort established from gene expression data of RCC tumor samples in TCGA cohort. Genes ultimately incorporated into the model were designated as prognostic genes. An independent validation cohort was employed to assess the model’s reliability and generalizability. For external validation, we accessed an independent dataset from the University of California Santa Cruz (UCSC) Xena Genomics platform (https://xenabrowser.net/datapages/ (accessed on 25 June 2025)). Following data integration and quality control procedures, we retained 121 samples with comprehensive gene expression and clinical information from the Pan-Cancer Analysis of Whole Genomes (PCAWG) cohort. The independent PCAWG validation cohort (or PCAWG cohort) was chosen because it provided complete clinical data including OS, unlike other datasets (e.g., ICGC, GDC) with missing information. Additionally, by utilizing 121 previously unanalyzed RCC samples from the PCAWG cohort, we effectively circumvented sample overlap concerns that could arise when using other datasets in conjunction with TCGA samples. Furthermore, the PCAWG cohort featured uniformly processed gene expression data, ensuring analytical consistency.

To validate the transcriptome data from the TCGA training cohort, we extracted the mRNA and protein expression profiles of candidate core genes and prognostic genes in 35 RCC-related tumor cell lines from the Human Protein Atlas (HPA, https://www.proteinatlas.org/ (accessed on 25 June 2025)), database. These data exhibit high homogeneity and robust reproducibility across RCC-related tumor cell lines, meeting the necessary standards for rigorous in vitro validation.

### 2.2. Identification of Differentially Expressed Genes

We performed differential expression analysis of the 4817 SUMOylation-associated genes. Genes meeting the criteria of a false discovery rate (FDR) < 0.05 and an absolute log2-fold change (|log2FC|) > 1 were defined as differentially expressed genes (DEGs). After removing genes without unambiguous Ensembl ID annotations, 298 significant protein-coding DEGs were retained for subsequent analyses. Gene Ontology (GO) and Kyoto Encyclopedia of Genes and Genomes (KEGG) enrichment analyses were conducted using the clusterProfiler R package, with the complete annotated protein-coding genome used as the background reference and an FDR threshold of <0.05 for significance.

Because DEGs identification based on three-way subtype comparisons may be affected by inter-subtype heterogeneity and may generate offset expression patterns that require additional pathological stratification for clinical interpretation, a four-way Venn intersection analysis was subsequently performed. The resulting 151 overlapping DEGs were defined as RCC-specific DEGs, representing molecular features consistently shared across the TCGA-RCC cohort while minimizing the influence of subtype-dependent transcriptional variation.

Integrating Venn analysis, enrichment analyses based on foreground query gene set versus genome-wide reference background universe (FG vs. BG) statistical testing with an FDR threshold of <0.001, and consensus clustering analysis, we further identified 27 key DEGs potentially involved in SUMOylation-related processes in RCC. This systematic filtering strategy excluded thousands of genes from the initial 4817-gene candidate pool that lacked significant RCC-associated differential expression or robust enrichment in SUMOylation-related pathways, thereby removing genes with only weak or indirect associations with SUMOylation. To further explore functional interactions among these genes, we constructed a protein-protein interactions (PPI) network using the STRINGdb R package.

### 2.3. Establishment and Validation of a Prognostic Model for Sumoylation-Associated Genes

To evaluate the prognostic relevance of SUMOylation-associated genes, univariate Cox proportional hazards regression analysis was initially performed on the 27 RCC-specific SUMOylation-associated DEGs using the survival R package in a pooled cohort of stage I–IV RCC patients. Among these candidates, *GNA11* did not reach statistical significance in the overall RCC cohort but demonstrated prognostic relevance in patients with advanced-stage RCC (stage III–IV). Given its potential biological importance in advanced disease and its contribution to the SUMOylation-associated regulatory network, *GNA11* was retained together with the other five candidate genes for subsequent model refinement using least absolute shrinkage and selection operator (LASSO) regression.

LASSO regression analysis was performed using the glmnet R package for feature selection and model construction. Ten-fold cross-validation was applied to determine the optimal penalty parameter (λ), with the λ value corresponding to the minimum cross-validation error selected for subsequent model development. The TCGA cohort was exclusively used for model training and parameter optimization, whereas the independent PCAWG cohort (*n* = 121 RCC patients) was completely separated from the training process and used only for external validation. Subtype-stratified analyses of KIRC, KIRP and KICH were additionally performed to evaluate model robustness across different RCC subtypes.

Before prognostic model construction, the proportional hazards assumption was evaluated using Schoenfeld residual-based testing for the selected six signature genes and clinical covariates, including age, sex, tumor stage, and histological subtype. Variables with *p* > 0.05 in the proportional hazards test were considered consistent with the assumption. Several strategies were implemented to minimize potential overfitting. First, candidate genes were subjected to stepwise filtering through differential expression analysis, SUMOylation-related functional enrichment, protein–protein interaction analysis, univariate Cox regression, multivariate Cox regression, and LASSO regularization. Second, LASSO penalization reduced model complexity by shrinking coefficients of less informative variables. Third, the predictive performance and generalizability of the final model were assessed using an independent PCAWG validation cohort. Finally, 1000 bootstrap resampling iterations were performed for internal bias correction, and model performance was further evaluated using calibration curves, decision curve analysis (DCA), and time-dependent concordance index (C-index) analysis to assess calibration, clinical utility, and long-term discrimination.

Next, we standardized the gene expression data of the TCGA training cohort and calculated risk scores for each patient via the formula $Risk\;score=\sum \left({coef}_{i}\cdot {gene}_{i}\right)$, where ${coef}_{i}$ represents the regression coefficient and where ${gene}_{i}$ denotes the expression level of each SUMOylation-associated gene. On the basis of the median risk score, we stratified the 758 tumor samples into high- and low-risk groups. Using this risk stratification, we conducted further analyses, including survival analysis via the survminer R package, a 5-year receiver operating characteristic (ROC) curve was generated using the timeROC R package to evaluate predictive performance, and principal component analysis (PCA) via the FactoMineR and factoextra R packages. We validated the prognostic model in the PCAWG cohort via identical analytical pipelines, including normalization and risk score calculation, on the basis of the six-gene signature. All validation analyses, including risk score analysis, were conducted using the six previously identified prognostic signature genes.

### 2.4. Tumor Immune Microenvironment Analysis

We characterized the TIME by evaluating immune cell infiltration, gene set variation analysis (GSVA) was applied to the expression matrix of the SUMOylation-related gene set from 758 tumor samples. The analysis was conducted on a normalized gene expression matrix (log2-transformed TPM values) comprising all detected genes across 758 tumor samples, which had been previously stratified into high- and low-risk groups based on the median risk score derived from our prognostic signature. To identify differences in immune cell infiltration between risk groups, we performed Wilcoxon rank-sum tests. The results were visualized using (1) boxplots generated with the ggplot2 R package to display the distribution of immune cell proportions, and (2) clustered heatmaps created with the pheatmap R package to illustrate sample-level immune cell abundance profiles.

### 2.5. Immunohistochemical Analysis and Systematic Screening of Cellular Models and Target Genes

To further validate the clinical utility of 10 DEGs identified as core markers through RCC screening (based on gene expression matrices) and the 6 prognostic signature genes in our prognostic risk model, we retrieved immunohistochemical (IHC) staining data for these genes from the HPA database. For sample selection, we prioritized tumor tissues from individuals aged approximately 60 years, ensuring gender consistency, as KIRC cases aged ≥ 60 years constitute 60.03% of the cohort, with 55–65 years accounting for 32.32%.

Cell lines and target genes were screened using primary data from the HPA database (https://www.proteinatlas.org/humanproteome/cell+line/kidney+cancer (accessed on 25 June 2025)), which includes 35 RCC-derived tumor cell lines with comprehensive mRNA (normalized transcripts per million, nTPM) and protein expression profiles. Protein expression levels were quantified via mass spectrometry-based proteomics from the Pan-Cancer Atlas project, reported as normalized relative protein expression (nRPX) values with log2FC.

## 3. Results

### 3.1. Analysis of Differentially Expressed Genes

**Figure 1:**
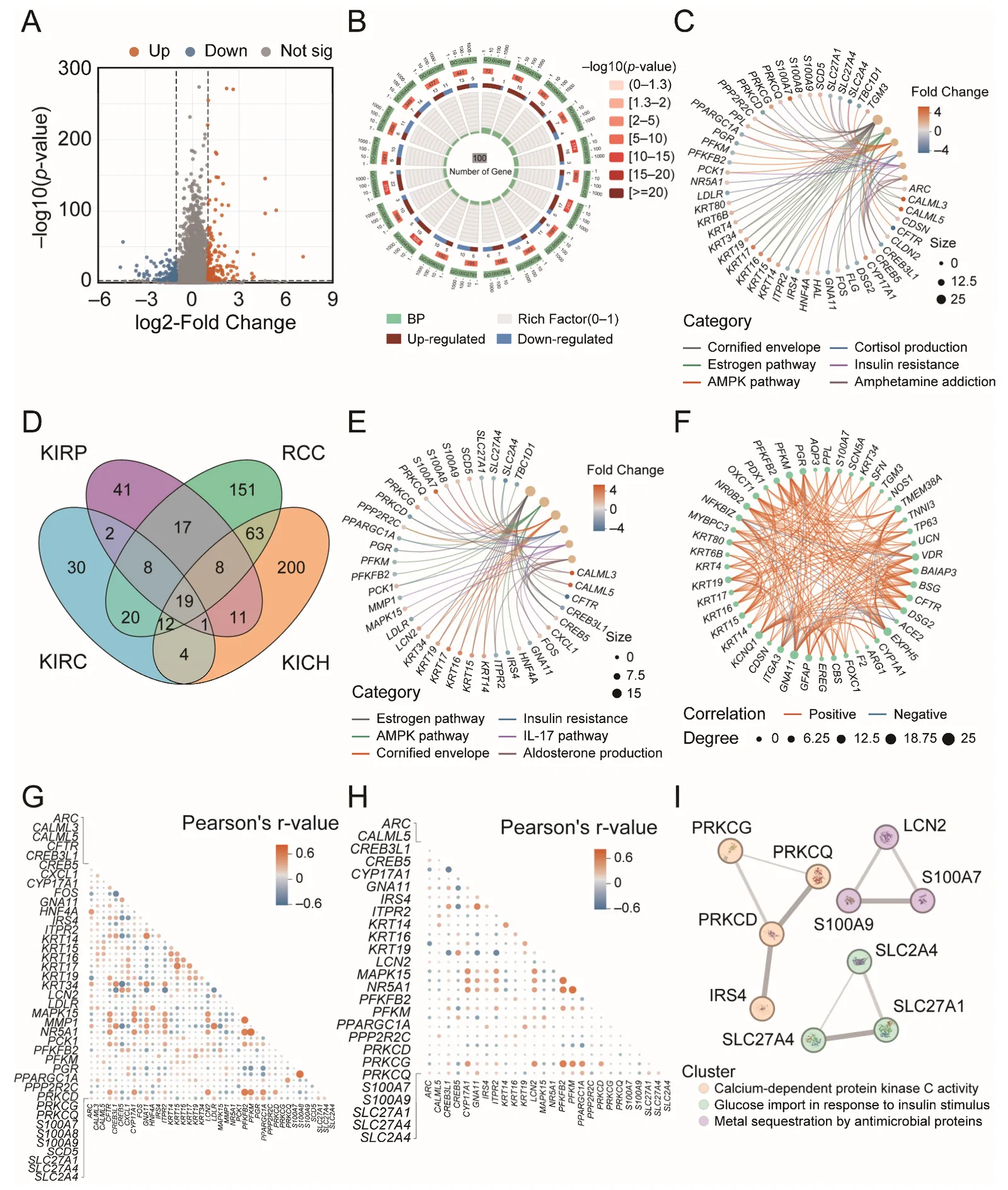
Systematic characterization of SUMOylation-associated DEGs in RCC. (**A**) Volcano plot depicting 298 significant SUMOylation-associated DEGs (red: upregulated; blue: downregulated). (**B**). Circos plot of the results of the GO enrichment analysis, highlighting the top enriched biological processes (BP). (**C**). KEGG pathway analysis showing six significantly enriched signaling pathways. (**D**). Venn diagram identifying 151 RCC-specific DEGs after excluding subtype-associated variants. (**E**). Consistency validation through secondary KEGG enrichment analysis of RCC-specific DEGs. (**F**). Correlation network of DEGs within the top 10 GO terms (red: positive; blue: negative). (**G**). Coexpression heatmap of the initial 298 DEGs (significant gene pairs (*p* < 0.05) are shown). (**H**). Coexpression heatmap of the 151 RCC-specific DEGs (significant gene pairs (*p* < 0.05) are shown). (**I**). PPI network of 10 core genes clustered into three functional modules: calcium-regulated protein kinase C signaling (orange), insulin-responsive glucose transport (green), and antimicrobial metalloprotein activity (purple). Line thickness represents the strength of correlation.

An initial list of 4817 SUMOylation-associated genes was obtained from GeneCards. Based on this list, gene expression profiles and clinical data for 891 RCC patients and 129 normal individuals were retrieved from TCGA. Differential expression analysis using stringent criteria (FDR < 0.05 and |log2FC| > 1) revealed 298 DEGs, including 165 upregulated and 133 downregulated genes (Figure 1A). GO enrichment analysis (Figure 1B) of significantly enriched terms (FDR < 0.001) highlighted key biological processes such as epidermal morphogenesis, secretory pathways, metabolic regulation, protein localization, amide transport, and myofibril assembly. Among these, epidermal morphogenesis, protein localization regulation, and amide transport showed particularly strong enrichment, each with a gene enrichment ratio exceeding 7%. KEGG pathway analysis (Figure 1C) identified six dominant signaling cascades including 41 DEGs (13.76%).

Independent differential expression analyses were performed for the three major RCC subtypes (KIRC, KIRP and KICH), followed by a four-set Venn intersection analysis incorporating the 298 SUMOylation-associated DEGs identified from the pooled TCGA-RCC cohort (Figure 1D). Unlike conventional three-way intersection analysis, which primarily identifies genes showing consistent differential expression across all individual RCC subtypes, the four-set approach was designed to capture genes representing population-level molecular characteristics of RCC. This strategy accounts for the possibility that subtype-specific transcriptional variation may mask shared RCC-associated signals when individual subtypes are analyzed separately. The Venn analysis identified two distinct DEG groups: 19 genes shared among all four datasets and 151 genes specifically identified in the pooled TCGA-RCC cohort. The 19 shared DEGs exhibited significant differential expression across individual RCC subtypes as well as the overall RCC cohort; however, their expression patterns varied among subtypes. Such subtype-dependent expression variation may generate opposing signals when subtype cohorts are combined, reducing the robustness of tumor–normal discrimination without prior pathological stratification and limiting their utility to subtype-specific molecular analyses. In contrast, the 151 pooled-cohort-specific DEGs represented genes whose RCC-associated expression alterations were detectable at the population level but were not consistently identified within individual subtypes, suggesting that their signals may be obscured by inter-subtype heterogeneity. These genes therefore capture broader molecular characteristics of RCC independent of predefined pathological classification and may provide a more suitable foundation for developing a universal prognostic model applicable across RCC subtypes. Accordingly, these 151 genes were designated as RCC-specific DEGs in this study.

Among 151 RCC-specific DEGs (50.67%), the interleukin-17 (IL-17) signaling pathway and aldosterone synthesis/secretion had higher significance priority than cortisol synthesis and secretion and amphetamine addiction (Figure 1D,E). To further explore functional significance, we prioritized the top 10 GO biological processes based on the foreground query gene set vs. genome-wide reference background universe (FG vs. BG) ratio from the enrichment results (FDR < 0.001). Correlation analysis of DEGs involved in these processes (Figure 1F) showed that the majority of gene pairs were positively correlated (*p* < 0.05), while the angiotensin-converting enzyme 2 gene *ACE2* exhibited consistent negative correlations with other DEGs (*p* < 0.05). Complementary heatmaps (Figure 1G,H) illustrated the interaction networks among genes in the six KEGG pathways. Positive co-expression patterns were predominantly observed within both the full 298-DEGs set and the RCC-specific 151-DEGs subset (Figure 1D, Supplementary Figure S1). However, no statistically significant differences were observed between the co-expression network architectures of the two groups (*p* > 0.05).

To identify core SUMOylation-associated DEGs, we performed iterative functional enrichment analysis (Supplementary Figure S2), which led to the identification of ten core genes (*PRKCG*, *PRKCQ*, *PRKCD*, *IRS4*, *SLC2A4*, *SLC27A1*, *SLC27A4*, *LCN2*, *S100A7*, and *S100A9*). PPI network analysis (Figure 1I) organized these core gene-associated proteins into three functionally distinct modules. Significant co-expression was observed within modules (*p* < 0.05), suggesting strong intramodular coordination of function.

### 3.2. Analysis of Differentially Expressed Genes at the Subtype Level

**Figure 2:**
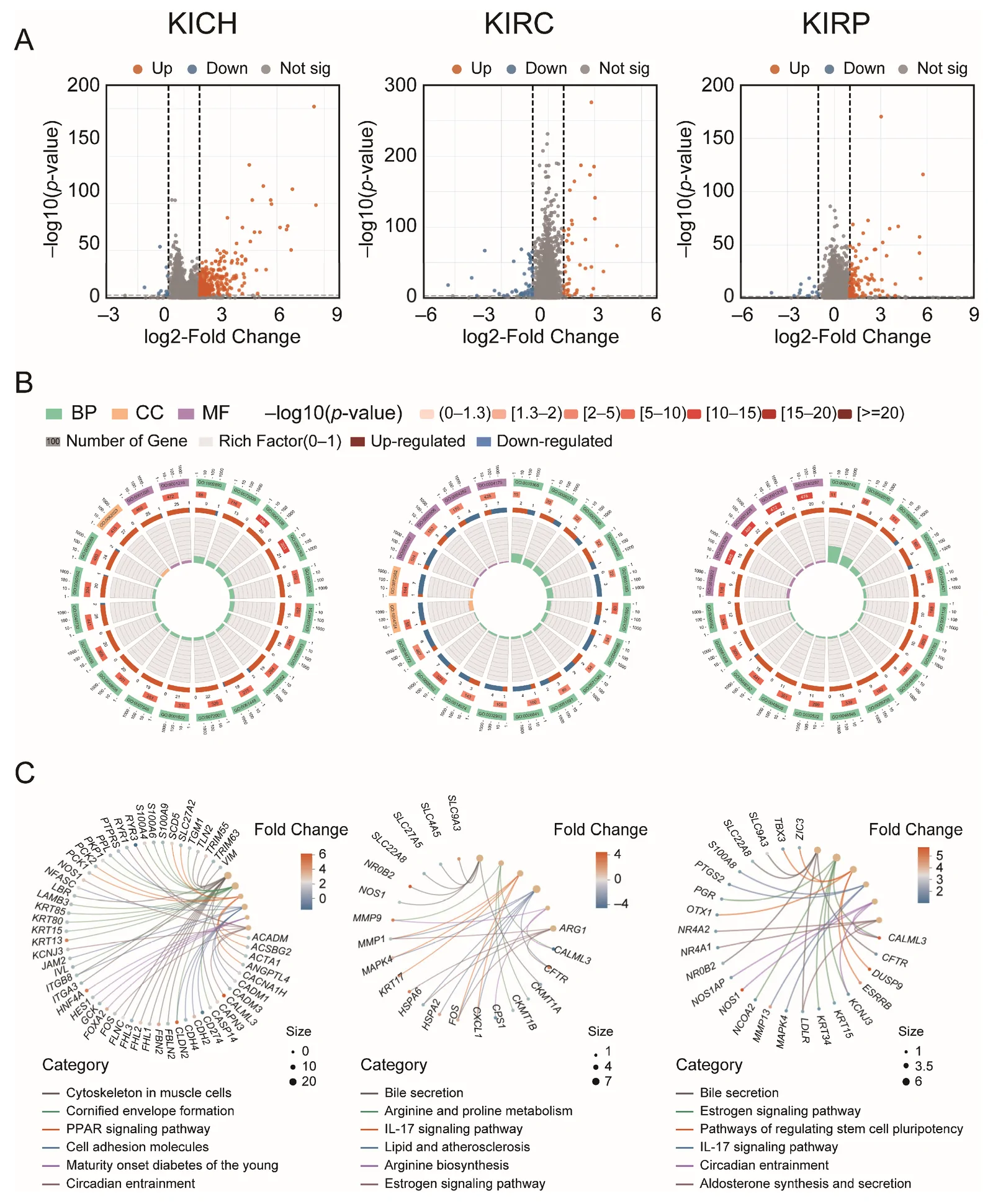
Identification of SUMOylation-associated DEGs in RCC subtypes from the TCGA training cohort. (**A**) Volcano plot showing the differential expression patterns of SUMOylation-associated genes among three RCC subtypes (KICH, KIRC and KIRP). (**B**) GO enrichment analysis of identified DEGs. BP, biological process; CC, cellular component; MF, molecular function. (**C**) KEGG pathway enrichment analysis of the identified DEGs.

Subtype-specific differential gene expression analyses (Figure 2A) showed that the majority of DEGs were upregulated in both KICH (95.9%; 305 of 318 DEGs) and KIRP (89.7%; 96 of 107 DEGs). In contrast, KIRC exhibited a more balanced distribution of expression changes, with 43.8% of DEGs upregulated and 56.2% downregulated. GO enrichment analysis of the top 20 biological processes (Figure 2B) revealed several shared terms across subtypes, including cell differentiation, tissue development, morphogenesis, and transcriptional regulation. KEGG pathway enrichment analysis (Figure 2C) demonstrated that at the RCC subtype level, KIRC and KIRP exhibited greater similarity, with bile secretion, estrogen signaling pathway, and IL-17 signaling pathway identified as common pathways shared between the two subtypes. Despite this overlap, each subtype demonstrated distinct enrichment patterns (*p* < 0.05).

**Figure 3:**
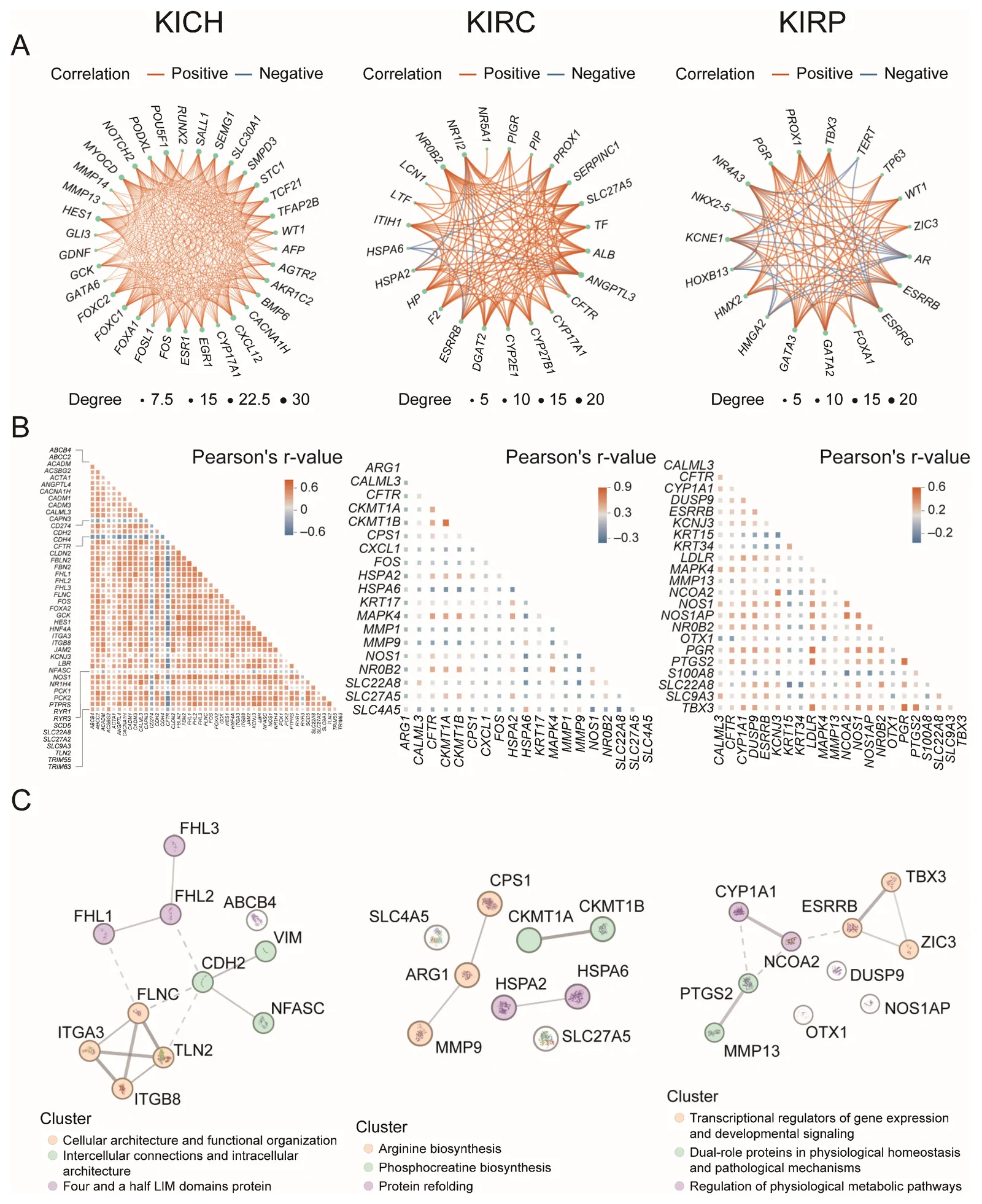
Correlation and PPI analyses of DEGs in RCC subtypes. (**A**) Interaction network of characteristic DEGs across three RCC subtypes (KICH, KIRC, and KIRP). (**B**) Correlation analysis of characteristic DEGs among the three subtypes (*p* < 0.05). (**C**) PPI network of key DEGs identified in the three subtypes. Line thickness represents the strength of correlation, with the dotted line indicating relatively weak associations.

To characterize subtype-specific heterogeneity in RCC, we systematically analyzed co-expression patterns of SUMOylation-associated DEGs constructed PPI networks for each subtype. The vast majority of subtype-specific DEGs demonstrated significant positive coexpression (*p* < 0.001), with bidirectional correlations showing stronger positive trends (Figure 3A). Strikingly, clinically relevant genes such as *CD274* (PD-L1, programmed cell death ligand 1, an immune checkpoint molecule), *CFTR* (a chloride channel regulator) and *RYR3* (a gene regulating calcium ion channels) displayed consistent negative correlations with KICH-specific DEGs (*p* < 0.05; Figure 3B, Supplementary Figure S3). To identify SUMOylation-associated genes, we first identified and analyzed DEGs on Venn diagrams of subtype-specific DEGs, followed by cross-subtype comparative profiling (Supplementary Figure S1). Additionally, we conducted secondary GO enrichment analysis specifically for KICH subtypes to further elucidate their unique molecular characteristics (Supplementary Figure S2). Hierarchical clustering of key DEGs (Figure 3C) revealed three functionally distinct modules per subtype, with limited intermodule connectivity.

### 3.3. Consensus Clustering and Expression Profile Analysis of TCGA-RCC Samples

**Figure 4:**
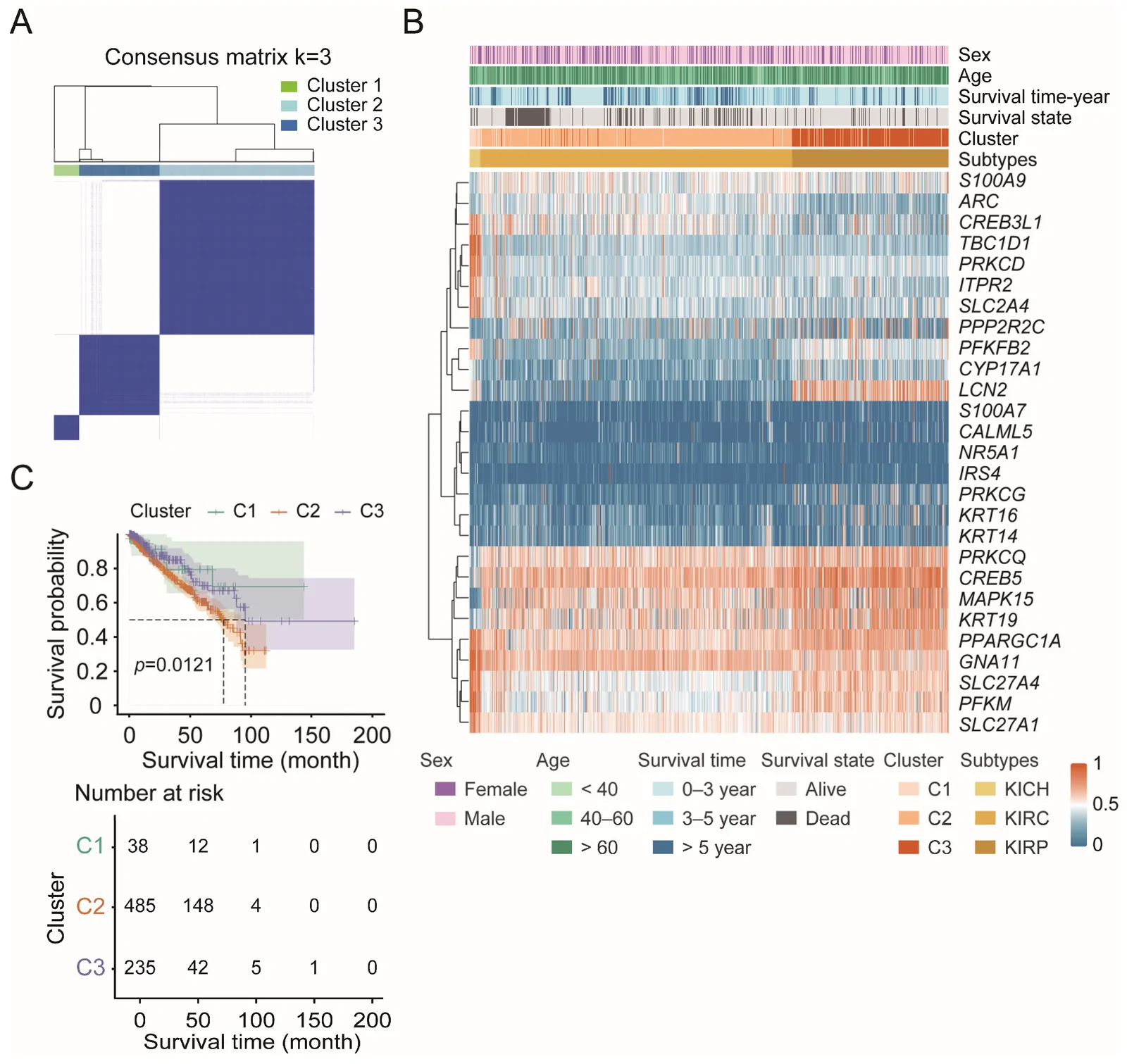
Molecular subtyping and survival analysis of the TCGA-RCC cohort on the basis of 151 SUMOylation-associated DEGs. (**A**) Consensus clustering of RCC tumor samples. (**B**) Hierarchical clustering heatmap of 27 key DEGs in tumor samples. (**C**) Kaplan-Meier survival analysis of tumor subtypes defined by consensus clustering.

To elucidate the underlying associations between RCC subtypes and DEGs implicated in SUMOylation, we employed consensus clustering on a cohort of 758 RCC samples from TCGA training cohort, utilizing the expression profiles of 151 DEGs (Figure 4A). 27 key DEGs were identified through enrichment analysis (Supplementary Figure S1). While no significant associations were observed between these clusters and age, survival status, or OS, the molecular classification demonstrated remarkable consistency with established RCC subtypes from the TCGA database (Figure 4B).

Kaplan-Meier (K-M) survival analysis of the three molecular clusters revealed significant prognostic value (*p* = 0.0121; Figure 4C). Cluster 1 (C1, predominantly KICH) maintained superior survival outcomes, with 10-year OS rates exceeding 70%. The median OS for C2 (predominantly KIRC) and C3 (primarily KIRP) patients reached 80 and 95 months, respectively. At the 50-month follow-up, the risk table indicated that 31.6% (C1), 30.5% (C2), and 17.9% (C3) of the original participants remained in each respective group. This finding indicates that in the current TCGA training cohort, C3, which is predominantly composed of KIRP, has a higher mortality rate than C1 and C2.

### 3.4. Prognostic Model Development and Validation in TCGA-RCC Samples

**Figure 5:**
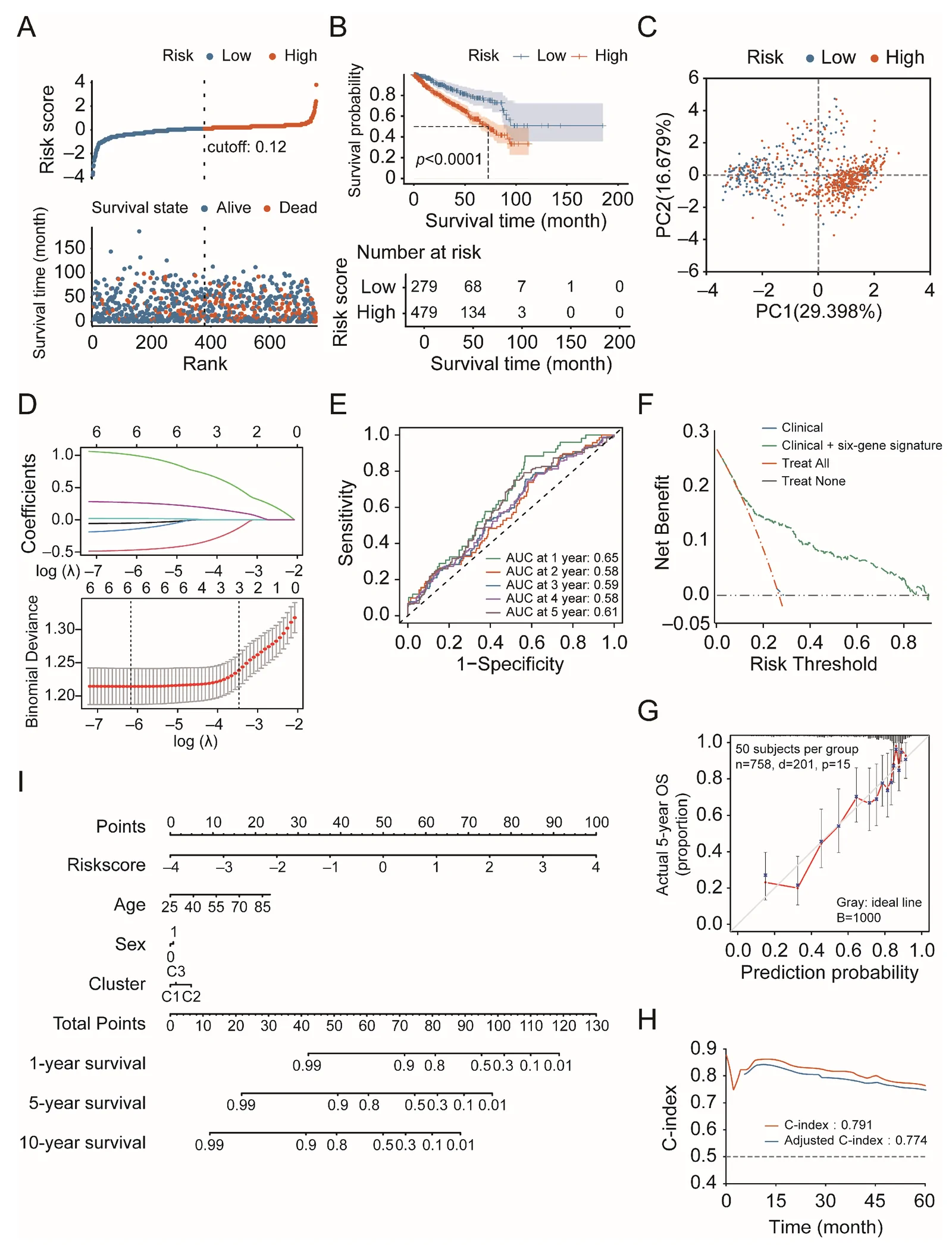
Development and validation of a SUMOylation-associated gene prognostic signatures in the TCGA-RCC cohort. (**A**) Risk score distribution and patient stratification based on the 27 SUMOylation-associated signatures. (**B**) Kaplan-Meier survival curves comparing the high- and low-risk groups. (**C**) PCA of tumor samples in the high-risk and low-risk groups. Number of tumor samples in the high-risk and low-risk groups over survival. (**D**) LASSO coefficient profiles of the 27 key genes and the final 6-gene selection by 10-fold cross-validation. (**E**) Time-dependent ROC curve analysis for predicting the 5-year survival rate. (**F**) Decision curve analysis for 5-year overall survival prognostic models. Black dashed line: no intervention (Treat None); red dashed line: universal intervention (Treat All). Light blue curve: clinical model; green curve: combined model with clinical variables plus six SUMOylation-related gene signatures. (**G**) Calibration curve of the prediction model for 5-year overall survival (OS). The cohort contained a total of 758 patients, of whom 201 developed OS events (death). The model incorporated 15 predictors, and each stratified subgroup included roughly 50 individuals. Gray line: ideal prediction. Red dots: raw survival; blue crosses: bias-corrected values from 1000 bootstrap resamples. Vertical bars: confidence intervals. (**H**) Time-dependent C-index curve of the combined model (60 months). Gray dashed line: discrimination threshold of 0.5. Red: raw C-index = 0.791; blue: bootstrap-adjusted C-index = 0.774. (**I**) Clinical nomogram integrating the gene signature with age, sex and tumor stage.

We developed a prognostic signature via risk analysis, which stratified patients into high-risk (*n* = 479) and low-risk (*n* = 279) groups (Figure 5A). Comprehensive validation analyses were conducted, including K-M survival comparison, centroid-based PCA for dimensionality reduction, LASSO regression for feature selection, and time-dependent ROC analysis for predictive accuracy assessment (Figure 5B–E). Survival analysis revealed striking differences between risk groups (Figure 5B, *p* < 0.0001). Centroid PCA revealed that high-risk samples clustered along the positive half-axis of PC1, indicating that PC1 plays a key role in sample differentiation (Figure 5C).

LASSO regression refined the 27 candidate DEGs into a six-gene prognostic signature comprising *CREB3L1*, *GNA11*, *PFKM*, *PPARGC1A*, *PPP2R2C*, and *PRKCG* (Figure 5D). Stage-stratified Cox regression analysis revealed stage-dependent heterogeneity in the prognostic contribution of *GNA11*. In the overall RCC cohort consisting of stage I–IV patients (Table 1), *GNA11* showed a borderline but non-significant association with overall survival (*p* = 0.08). In contrast, when the analysis was restricted to patients with advanced RCC (stage III–IV), *GNA11* emerged as a significant independent protective factor (*p* < 0.01, Supplementary Table S1). These findings indicate that the prognostic relevance of *GNA11* is predominantly associated with advanced RCC and may be influenced by disease-stage composition in the pooled cohort. The absence of statistical significance in the overall cohort likely reflects stage-dependent variation in *GNA11*-associated survival effects rather than a lack of biological relevance. The prognostic risk score was calculated as follows:

$$Risk\;Score=(-0.0595\cdot CREB3L1)+\left(-0.4870\cdot GNA11\right)+\left(1.0606\cdot PFKM\right)+\left(-0.1882\cdot PPARGC1A\right)+\left(0.0211\cdot PPP2R2C\right)+\left(0.2814\cdot PRKCG\right).$$

Time-dependent ROC curve analysis demonstrated stable predictive accuracy (5-year AUC = 0.61) across follow-up periods (Figure 5E). Based on these findings, we performed multidimensional validation analyses, including decision curve analysis (DCA), calibration assessment, time-dependent C-index evaluation, and bootstrap resampling, to further evaluate the predictive performance and clinical applicability of the prognostic model. DCA demonstrated that the six-gene signature provided additional clinical net benefit beyond conventional clinical indicators, while the integrated model achieved superior risk stratification performance and potential utility for individualized treatment decision-making (Figure 5F). The 5-year calibration curve (Figure 5G) showed close agreement between predicted and observed survival probabilities, with bootstrap-corrected estimates demonstrating stable model calibration. Time-dependent C-index analysis with bootstrap correction (Figure 5H) yielded a raw C-index of 0.791 and an optimism-adjusted C-index of 0.774. Notably, the adjusted C-index remained above 0.75 throughout the 3–60 month follow-up period, indicating sustained and robust discrimination ability over time.

After adjustment for established clinical covariates, multivariate Cox proportional hazards regression further confirmed the independent prognostic value of the six-gene signature (Table 2). The calculated risk score remained an independent predictor of overall survival (HR = 1.31, 95% CI: 1.02–1.69, *p* < 0.05). In addition, advanced tumor stage and older age (>60 years) were independently associated with poorer outcomes, whereas sex and RCC histological subtype showed no significant independent association with overall survival. Multivariate risk assessment incorporating clinical variables and molecular subtypes (Figure 5I) demonstrated strong prognostic discrimination. The integrated risk score stratified patients into distinct prognostic tiers: low-risk (total points < 50, 10-year OS > 80%), intermediate-risk (total points ≈ 80, 5-year OS > 30%), and high-risk (total points > 100, 1-year OS > 30%). This SUMOylation-based model provides robust predictive power for clinical decision-making.

### 3.5. Internal Validation of the Prognostic Models at the Subtype Level

**Figure 6:**
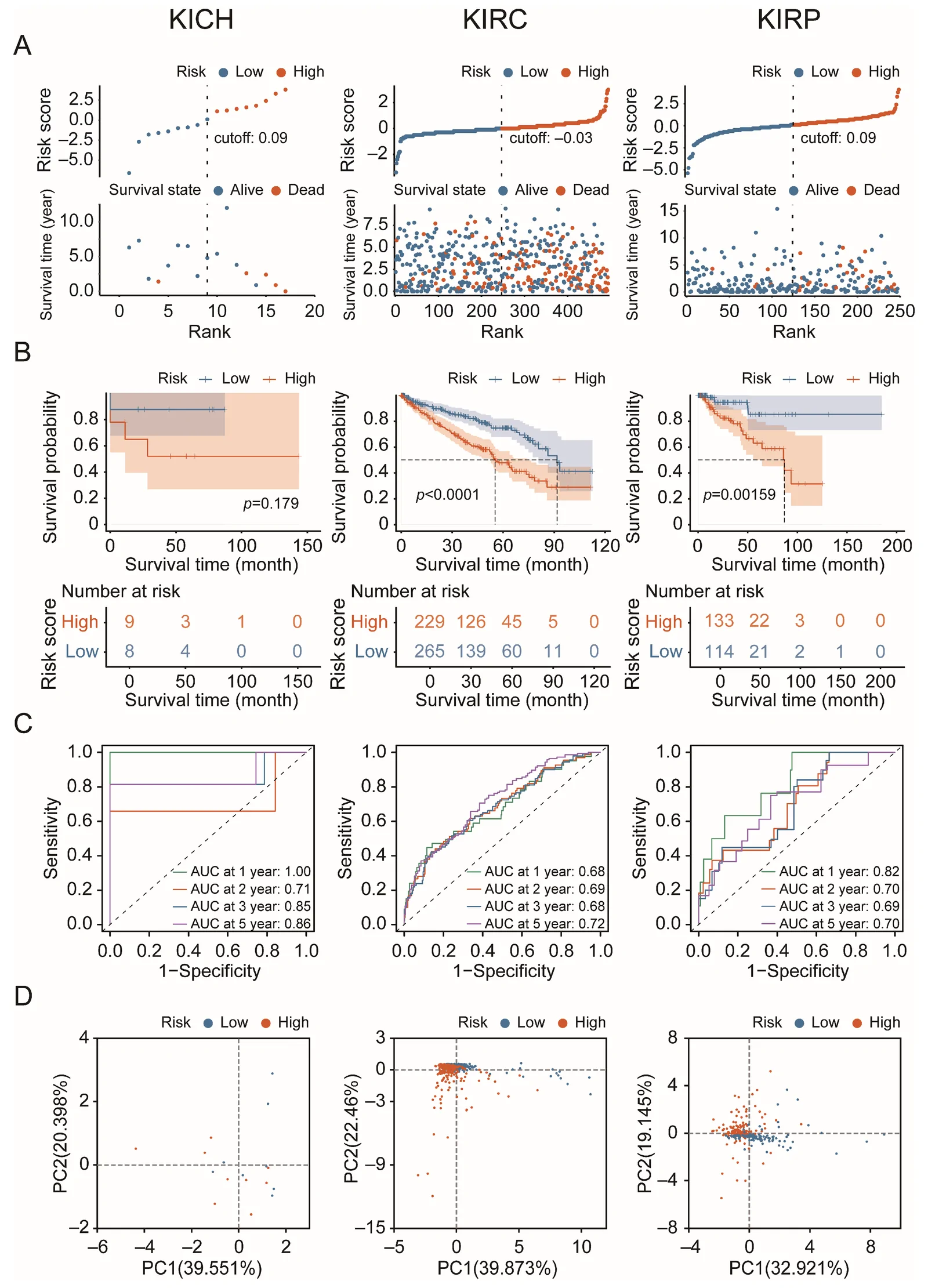
Validation of the SUMOylation-based prognostic model in RCC subtypes. (**A**) Risk score distribution across tumor subtypes. (**B**) Kaplan-Meier survival analysis of the high- and low-risk groups. (**C**) Time-dependent ROC analysis for 5-year overall survival prediction. (**D**) PCA of tumor samples stratified by risk group.

**Figure 7:**
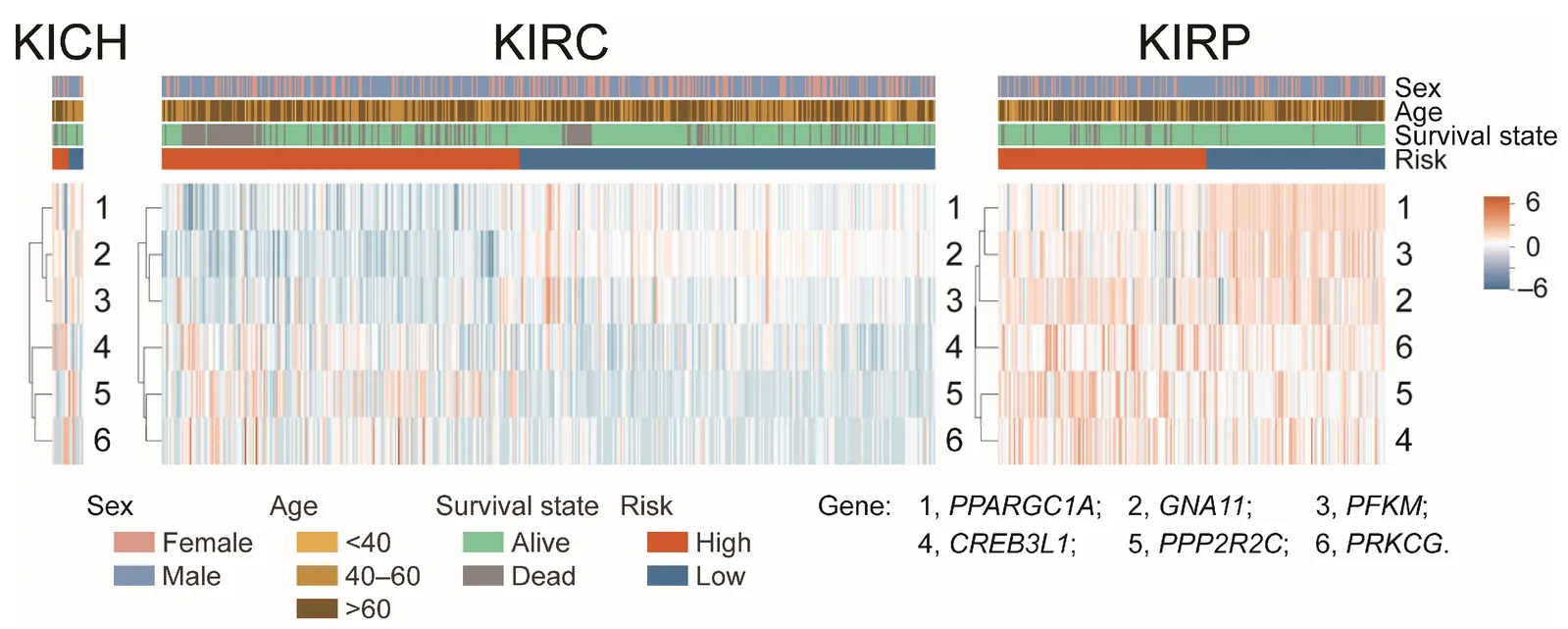
Expression heatmaps of six prognostic signature genes.

The TCGA-RCC cohort (*n* = 758) was classified into molecular subtypes (KICH = 17, KIRC = 494, KIRP = 247) per 2024 TCGA guidelines. Following normalization, we evaluated the performance of the six-gene prognostic signature across RCC subtypes. Previous studies have demonstrated that substantial molecular heterogeneity among RCC subtypes represents a major challenge for accurate tumor classification, targeted therapeutic development, and prognostic evaluation [13]. Therefore, to comprehensively investigate the applicability of the signature across the three major RCC subtypes (KICH, KIRC and KIRP), all available KICH cases (*n* = 17) were retained for subtype-stratified analysis. Although the limited sample size of the KICH cohort reduces statistical power and warrants cautious interpretation, these exploratory findings provide preliminary insights into the potential applicability of the prognostic signature in this less-represented RCC subtype and may inform future subtype-specific clinical investigations. The risk score analysis (Figure 6A) revealed that the proportions of high-risk samples in the three subtypes were 52.94% for KICH, 46.36% for KIRC, and 53.85% for KIRP. K-M analysis (Figure 6B) revealed significant prognostic stratification in KIRC (*p* < 0.0001) and KIRP (*p* = 0.0016), with median OS of 55 and 87 months, respectively. Time-dependent ROC analysis demonstrated consistent predictive accuracy across 5-year follow-up (AUC > 0.7), without significant temporal variation (Figure 6C). Centroid PCA (Figure 6D) demonstrated distinct distribution patterns of high- and low-risk samples across subtypes, with high-risk samples predominantly clustering on the negative side of PC1. Hierarchical clustering and heatmap analysis integrated expression patterns of the six prognostic signature genes with key clinical variables across three subtypes (Figure 7). The six-gene signature exhibited dichotomous prognostic associations: *PPARGC1A*, *GNA11* and *PFKM* were upregulated in low-risk samples, while *PRKCG*, *PPP2R2C* and *CREB3L1* showed reciprocal high-risk enrichment.

### 3.6. External Validation of the Prognostic Model in the PCAWG Cohort

**Figure 8:**
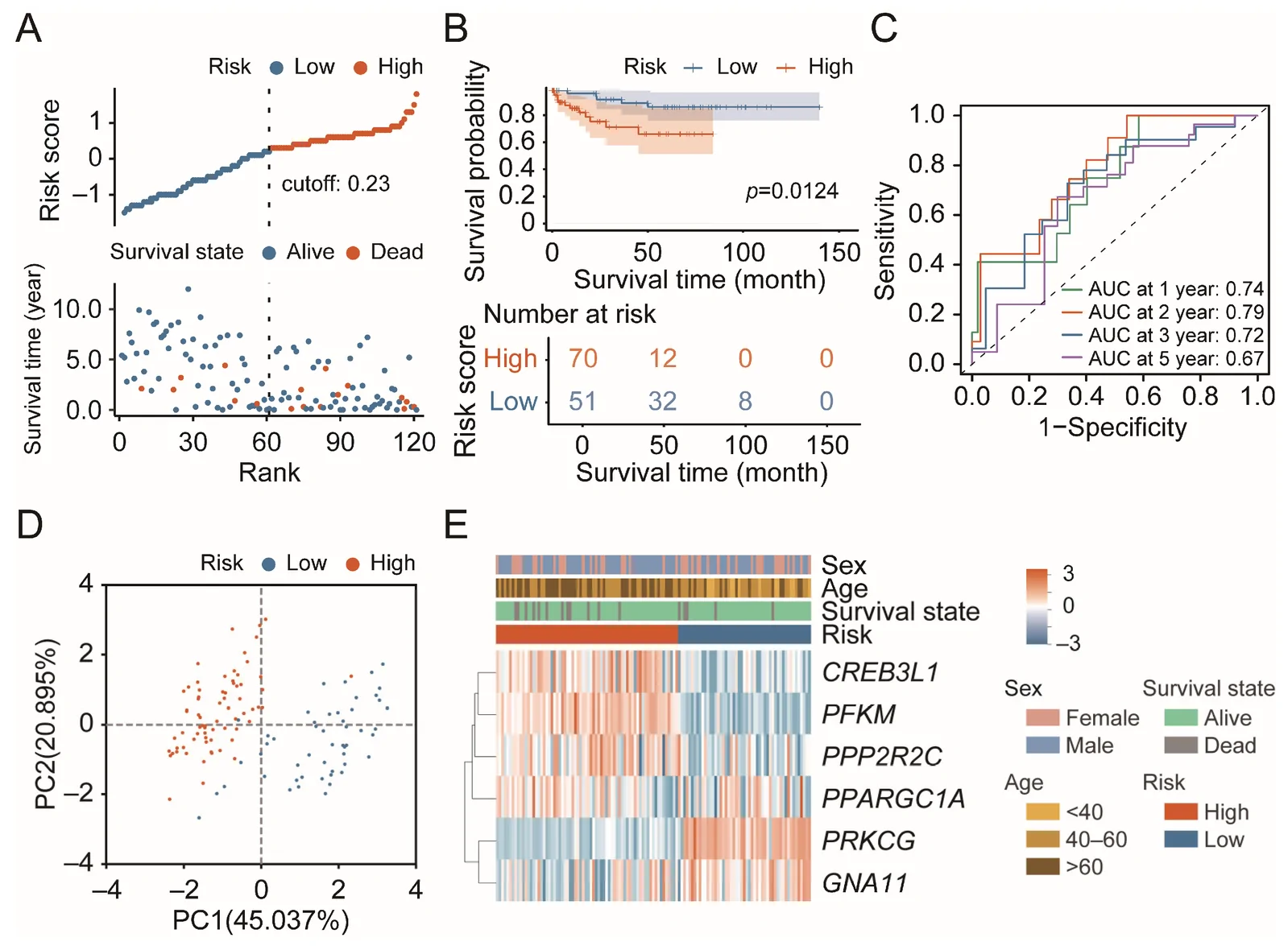
External validation of the RCC prognostic model using the PCAWG cohort. (**A**) Distribution of risk scores in the validation cohort. (**B**) Kaplan-Meier survival analysis between risk groups. (**C**) Time-dependent ROC analysis for 5-year overall survival prediction. (**D**) PCA of tumor samples stratified by risk group. (**E**) Expression heatmap of six prognostic signature genes with sample clustering.

For external validation, we analyzed 121 primary RCC samples from the PCAWG consortium (UCSC Xena release 2017) with matched whole-genome sequencing and clinical data. After identical preprocessing, we focused on the six prognostic signature genes to ensure consistency with the TCGA training cohort analysis. Using the established risk score cutoff, patients were stratified into high-risk (*n* = 70, 57.85%) and low-risk (*n* = 51, 42.15%) groups (Figure 8A). K-M survival analysis confirmed a significant difference between risk groups (*p* = 0.012, Figure 8B). Time-dependent ROC analysis (Figure 8C) demonstrated robust discriminative ability (3-year AUC = 0.72), meeting clinical utility thresholds. We performed hierarchical clustering and heatmap analysis using gene expression and clinical data. Centroid PCA (Figure 8D) revealed that high-risk samples were primarily distributed on the negative PC1 axis. The six prognostic signature genes were divided into two clusters (Figure 8E): Cluster 1 (*CREB3L1*, *PFKM*, *PPP2R2C*, *PPARGC1A*) and Cluster 2 (*PRKCG*, *GNA11*). This clustering pattern differed from that observed in the TCGA training cohort subtypes during internal validation.

### 3.7. Analysis of Immune Infiltration

**Figure 9:**
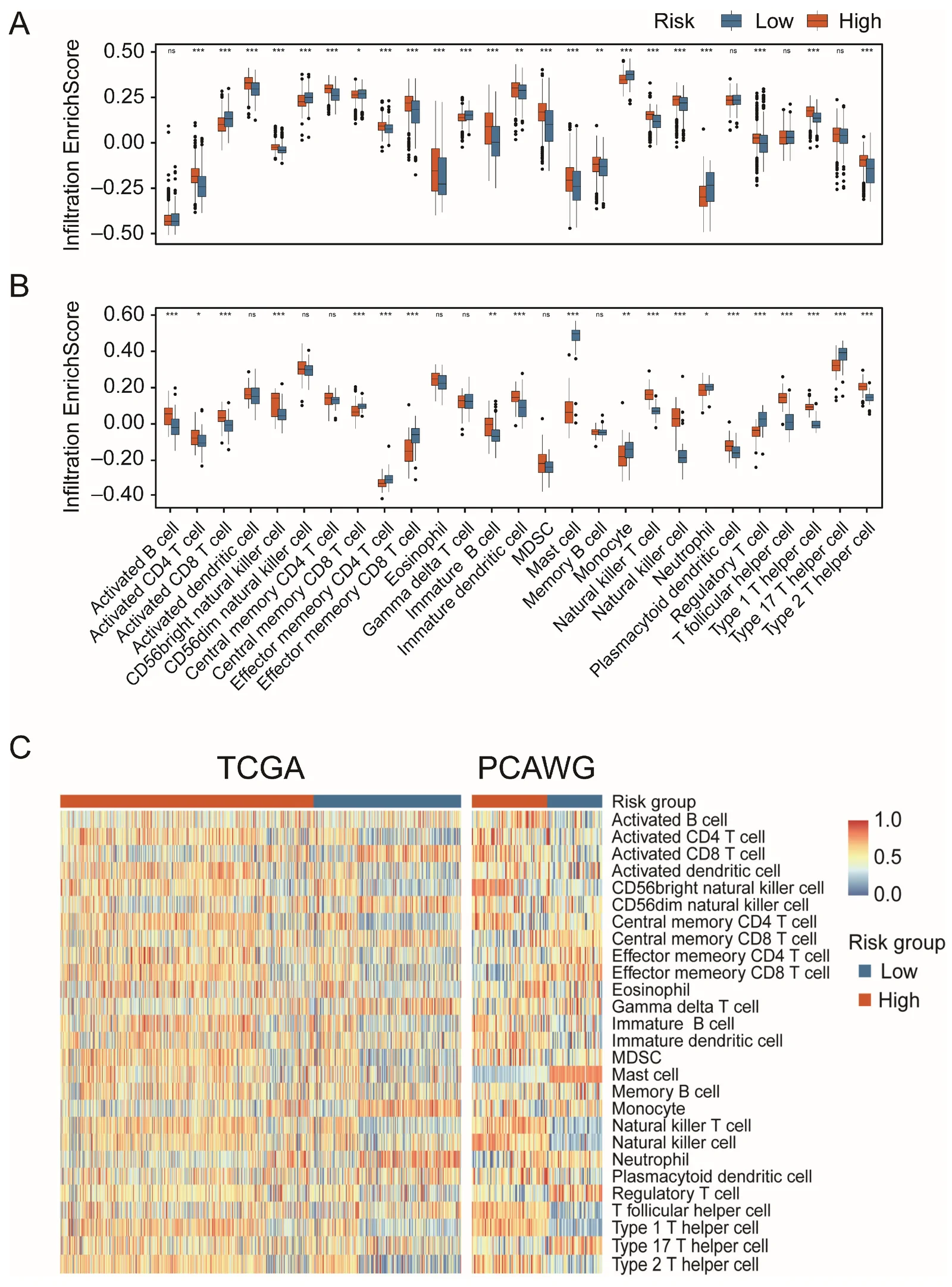
Immune infiltration analysis between risk groups in TCGA and PCAWG cohorts. Differences in immune cell infiltration between high- and low-risk groups in the TCGA (**A**) and PCAWG (**B**) cohorts. (**C**) Heatmap depicting differential immune infiltration patterns between risk groups across both cohorts. ns indicates *p* > 0.05; * indicates *p* < 0.05; ** indicates *p* < 0.01; *** indicates *p* < 0.001.

**Figure 10:**
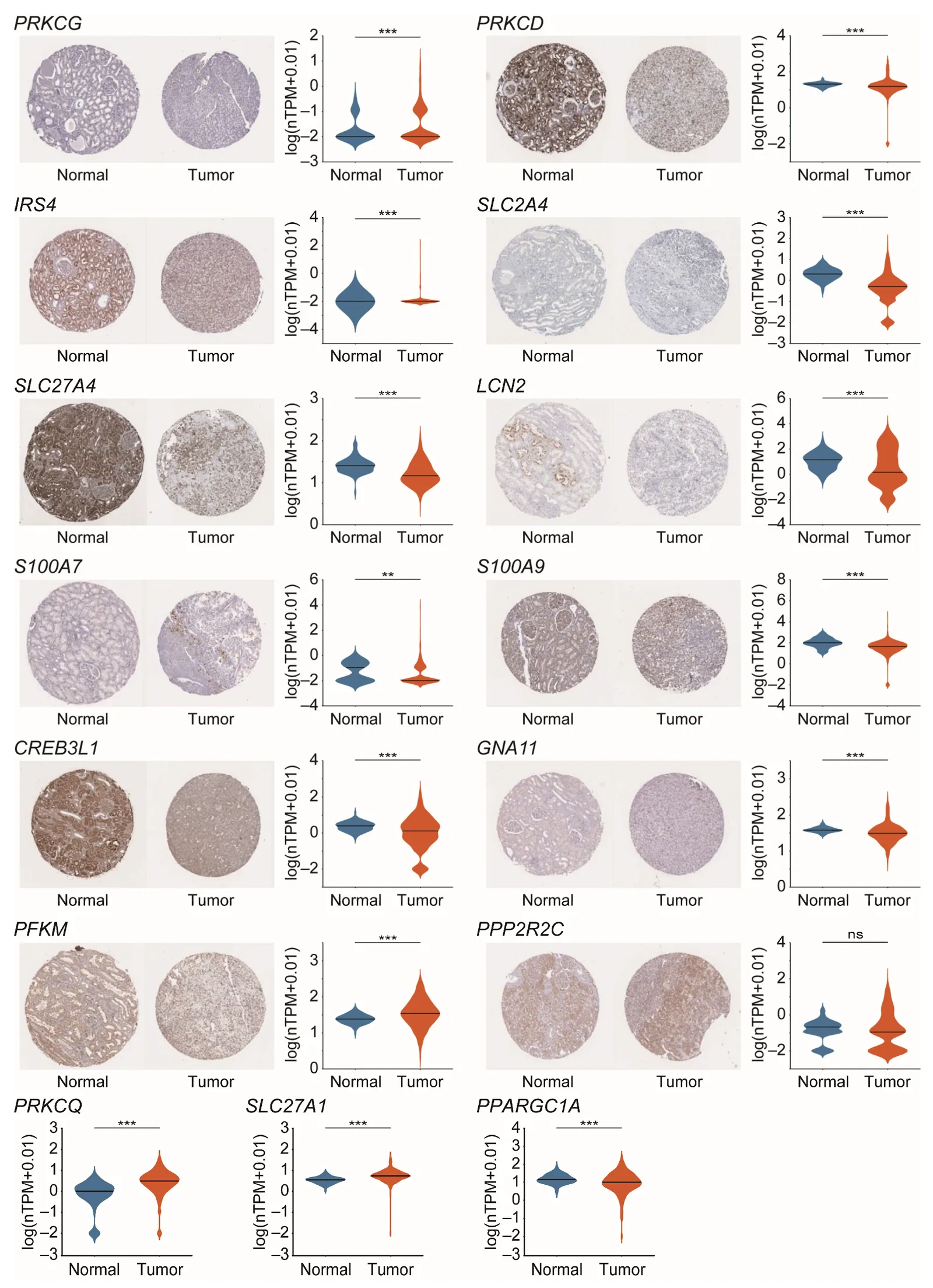
Immunohistochemical staining of SUMOylation-associated core DEGs and 6-prognostic signature genes in RCC. *PRKCQ*, *SLC27A1* and *PPARGC1A* are omitted due to unavailable immunohistochemical data from HPA database (https://www.proteinatlas.org (accessed on 25 June 2025)) [27]. ns indicates *p* > 0.05; ** indicates *p* < 0.01; *** indicates *p* < 0.001.

Immune infiltration patterns were analyzed in the TCGA and PCAWG cohorts to characterize tumor–immune interactions. In the TCGA dataset, 24 immune subsets (88.9%) showed significantly different abundances between risk groups (*p* < 0.05), with 20 subsets (74.1%) showing highly significant differences (*p* < 0.001; Figure 9A). The PCAWG cohort revealed consistent trends, with 16 subsets (59.3%) significantly altered (*p* < 0.001; Figure 9B), though with distinct cellular patterns. Heatmap analysis (Figure 9C) indicated higher infiltration of immune cells such as CD4^+^ T cells, dendritic cells, and NK cells in high-risk samples in TCGA. This trend was less consistent in PCAWG. Mast cells, however, were more abundant in low-risk groups. Subtype analysis (Supplementary Figure S4) showed that high-risk samples generally exhibited stronger immune infiltration across subtypes. To assess clinical relevance, we evaluated IHC expression patterns of core genes and model-related markers in RCC (Figure 10). Most core genes showed expression trends consistent with transcriptomic data. *PRKCD* and *SLC27A4* were more strongly expressed in normal tissues, suggesting potential tumor-suppressive roles, while *S100A7* was upregulated in tumors, indicating oncogenic potential. Among the prognostic model genes, *CREB3L1* showed tumor-suppressive expression patterns. In contrast, *PPP2R2C* and *PFKM* were more highly expressed in tumors.

### 3.8. Systematic Screening of Candidate Cellular Models and Key Target Genes

**Figure 11:**
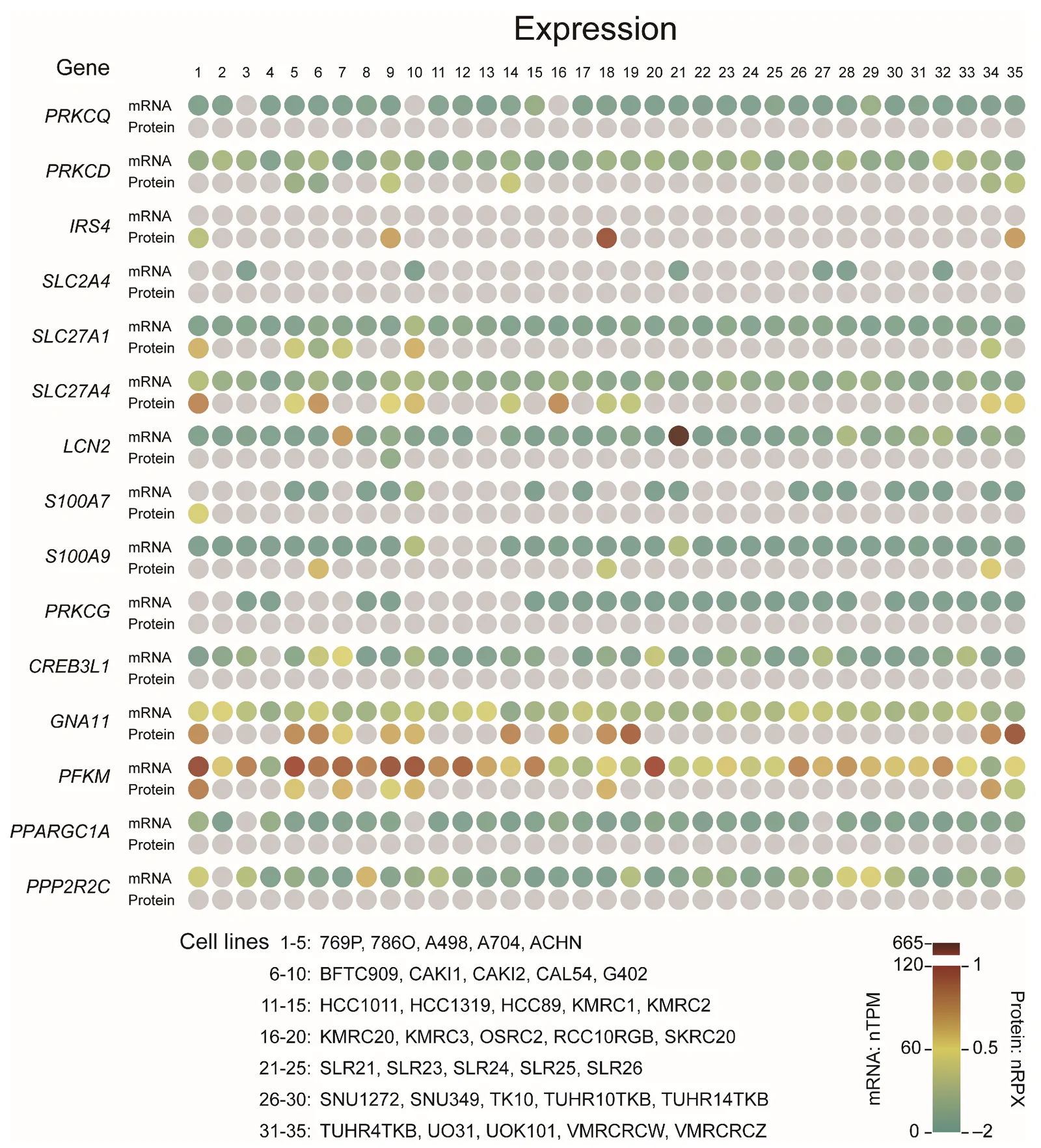
Expression of SUMOylation-associated and prognostic risk genes in RCC cell lines. mRNA (nTPM, normalized transcript per million) and protein (nRPX, normalized relative protein expression; log2-value) expression data are shown. Grey circles indicate undetected/missing data (Pan-Cancer Atlas MS data).

Through systematic evaluation of mRNA (nTPM, normalized transcripts per million) and protein (nRPX, normalized Relative Protein Expression, log2FC) expression profiles, we preliminarily identified seven optimal cell line models (CAL54, 769P, ACHN, BFTC909, CAKI1, G402, and the VMRCRCW/VMRCRCZ cell pair) and eight target genes (*PRKCD*, *SLC27A1*, *PFKM*, *SLC27A4*, *GNA11*, *PPARGC1A*, *PPP2R2C* and *LCN2*) for subsequent validation studies (Figure 11). This dual-omics approach enables reliable cross-validation of transcriptional and translational dynamics, thereby ensuring the selection of biologically representative research systems.

**Figure 12:**
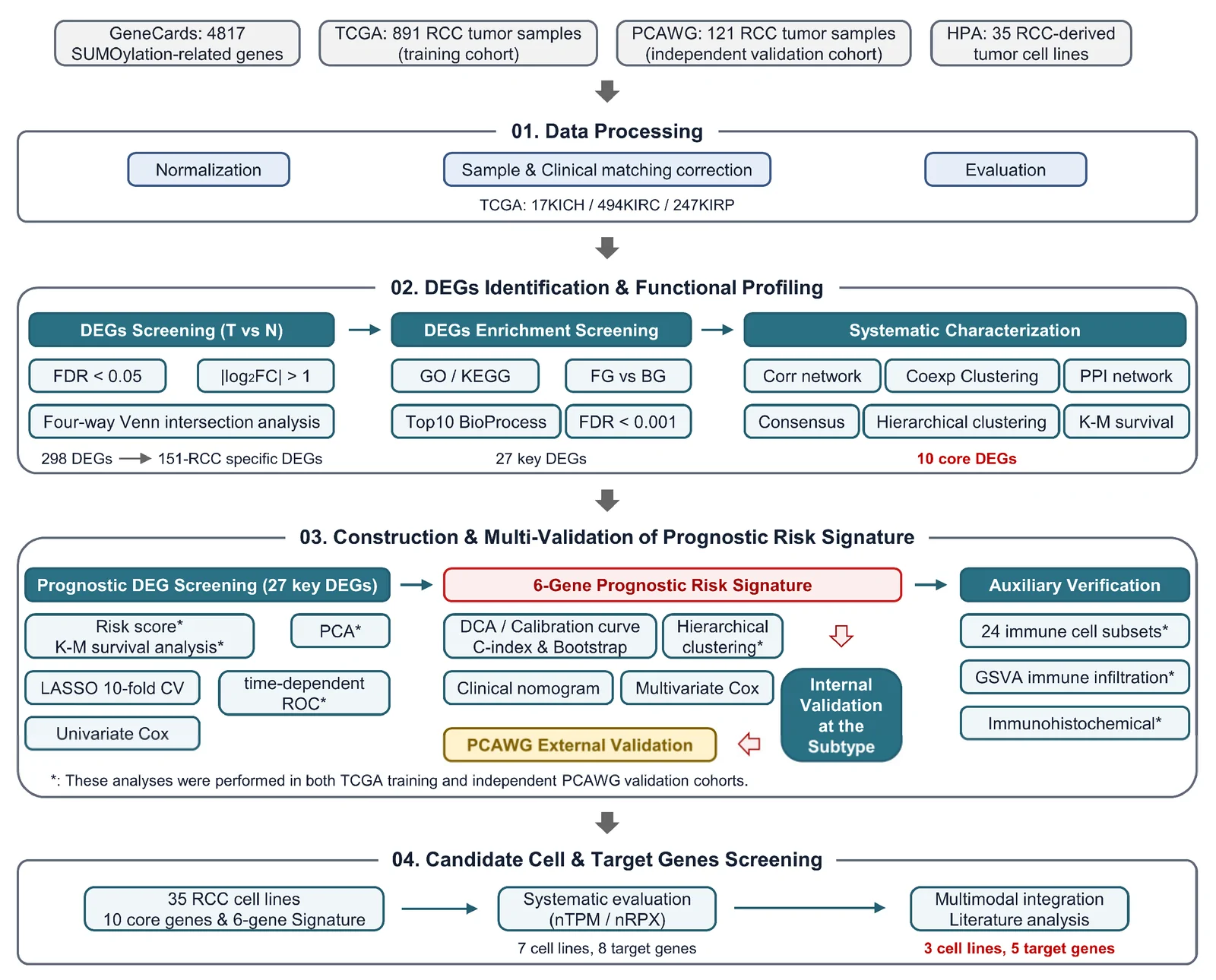
Schematic diagram of screening design and workflow.

Among the examined cell lines, *PRKCD*, *SLC27A1*, and *PFKM* exhibited widespread mRNA expression but displayed significant mRNA-protein discordance at the translational level (e.g., in ACHN, BFTC909, CAKI1, and CAL54 cell lines). Notably, *PFKM* maintained consistently high expression across multiple cell lines, making it an ideal candidate for investigating transcript-translation decoupling mechanisms. *SLC27A4* and *GNA11* demonstrated stable transcription-translation coupling in diverse cell lines, suggesting minimal regulatory interference from cellular contexts. This stability supports their potential as therapeutic targets or diagnostic biomarkers. Among other genes, *PPARGC1A* and *PPP2R2C* displayed broad dynamic ranges in mRNA expression (0 ≤ nTPM ≤ 68.7) across cell lines, reflecting their variable transcriptional states (from silencing to high activity). These genes may serve as valuable markers for studying cellular adaptability and disease progression. *LCN2* emerged as a unique case due to its extreme overexpression in SLR21 cells (nTPM = 662) and exclusive protein expression in CAL54 cells (nRPX = −1.579). Combined with prior analyses indicating *LCN2* as a signature gene for KIRC subtypes, these findings provide a novel entry point for investigating LCN2’s mechanistic role in RCC. By integrating mRNA–protein expression correlation analysis, immunohistochemical characterization, literature evidence, and multi-omics data (Figure 12), we identified three representative RCC cell lines (BFTC909, CAKI1, and CAL54) and five functionally relevant genes (*PRKCD*, *SLC27A1*, *SLC27A4*, *LCN2* and *GNA11*) as prioritized candidate models for further investigation of their underlying molecular mechanisms. This framework integrates quantitative expression analysis (including regulatory discordance and extreme outliers) with biological relevance assessment, providing a robust platform for mechanistic and translational research. The selected models will systematically elucidate how SUMOylation-associated genes govern the multi-dimensional progression of RCC.

## 4. Discussion

RCC is the predominant urinary system malignancy and exhibits distinct molecular subtypes (KIRC, KIRP, KICH) with characteristic genetic alterations [4,5,6]. KIRC demonstrates frequent VHL, activating hypoxia and angiogenesis pathways, while KIRP and KICH display unique driver genes and metabolic profiles [28,29]. These molecular differences underlie clinical heterogeneity and inform therapeutic strategies [28,30]. Thus, deciphering these mechanisms enables molecular subtyping, facilitates precision therapy development, and reveals tumor microenvironment heterogeneity, advancing personalized RCC management.

We identified 298 SUMOylation-associated DEGs in RCC (165 up-/133 down-regulated) functionally enriched in epidermal development, cellular transport/metabolism, and key signaling pathways (estrogen/AMP-Activated Protein Kinase (AMPK)/insulin). PPI analysis identified 10 core genes form three functionally coordinated modules that synergistically promote RCC progression and therapy resistance through their interconnected regulatory networks. The three functional modules demonstrate distinct pathological mechanisms in RCC. Calcium-dependent PKC regulators (*PRKCG/Q/D)* serve as prognostic biomarkers [31,32,33], with *PRKCQ* activating NF-κB/mTOR survival pathways [34] and *PRKCD* stabilizing HIF1α to promote hypoxic adaptation [35]. Insulin-responsive modules exhibit *SLC2A4* (GLUT4) downregulation in aggressive phenotypes [36], and *SLC27A1/4*-mediated fatty acid uptake linked to both lipid metabolism and PD-L1 upregulation [37,38]. Antimicrobial metalloproteins modulate the TIME through *LCN2*-mediated iron sequestration/M2-TAM polarization [39,40] and *S100A7/A9*-induced proinflammatory signaling via RAGE with MDSC recruitment [41,42]. Notably, cross-module interactions demonstrate coordinated pathological mechanisms in RCC. PKC signaling suppresses GLUT4 membrane localization to promote fatty acid oxidation, while *S100A9* activates *PRKCD* to enhance *LCN2* expression, forming a pathogenic feedforward loop. These findings suggest promising therapeutic strategies through combined PKC inhibitors, FATP antagonists or LCN2-neutralizing antibodies with immunotherapy. However, further investigation is needed to elucidate subtype-specific functions and resistance mechanisms of these modules.

Current research on precision therapy for RCC and its major subtypes still presents several limitations. Büttner et al.’s 174-gene signature prognostic model enables molecular subtyping, and its clinical utility in rapid diagnosis is constrained by high complexity [43]. Zhao and Guan et al., although revealing its potential in distinguishing tumor microenvironment phenotypes and predicting prognosis, focused solely on a single gene without constructing a prognostic model, potentially limiting its clinical applicability [44,45]. Although Fu et al.’s analysis of KLF family genes revealed the diagnostic and therapeutic value of KLF5, the absence of model construction and validation weakens the logical chain of conclusions [46]. Both Bian’s cuproptosis model and Zeng’s lncRNA model were validated only in KIRC, leaving their efficacy in other RCC subtypes unconfirmed [47,48]. Building upon these findings, our study specifically addresses the heterogeneity among RCC subtypes. KICH and KIRP showed predominant DEG upregulation (95.91% and 89.72%, respectively) versus balanced regulation in KIRC. While bile secretion was conserved, subtype-specific pathways emerged: peroxisome proliferators-activated receptors (PPARs) signaling in KICH, arginine metabolism in KIRC, and pluripotency regulation in KIRP. Functional clustering revealed distinct biological roles, from KIRC protein folding to KIRP extracellular matrix remodeling. Furthermore, our research provides a comprehensive overview of the differences in SUMOylation-related gene expression across three major subtypes of RCC. From this analysis, we identified 151 DEGs, particularly the *LCN2*. The expression of *LCN2* can not only distinguish tumors from normal tissues but also differentiate KIRC from the other two renal cancer subtypes (KICH and KIRP). Therefore, *LCN2* shows promise as a potential biomarker to assist traditional pathological diagnosis, a finding supported by the literature [49]. Additionally, a prediction risk model containing only six genes was developed, demonstrating stable performance across different renal cancer subtypes. This serves as a “universal” SUMOylation-related prognostic tool applicable to several subtypes of RCC. Finally, the study established a ready-to-use experimental research framework involving “three cell lines and five genes,” along with corresponding gene and protein data. Meanwhile, numerous studies have validated the reliability of these cell lines for RCC mechanistic investigations [50,51,52,53]. This provides a practical platform for subsequent mechanism exploration and therapeutic target validation.

Analysis of the TIME revealed significantly distinct infiltration patterns between risk groups. High-risk TCGA samples presented stronger immune infiltration with elevated B cells, dendritic cells and Th1/Th2 activity, suggesting a dysregulated proinflammatory response, whereas low-risk PCAWG samples presented enhanced mast cell activity. The paradoxical association between immune activation and poor prognosis warrants further investigation into functional cell states. In addition, consistently across all molecular subtypes, high-risk samples maintained elevated immune infiltration levels compared with their low-risk counterparts, which is supported by the literature [54,55].

In RCC, the upregulation of the glycolytic enzyme *PFKM* not only drives metabolic reprogramming and tumor growth through ALDOB interaction but also serves as a marker of poor prognosis [56]. Conversely, the mitochondrial regulator *PPARGC1A* is downregulated in RCC [57]. Its reduced expression contributes to lipid accumulation, therapy resistance, and immune modulation via a miR-199b-dependent mechanism [58].

Furthermore, the kinase *GNA11* is significantly downregulated, and its low expression correlates with poor survival, immune cell infiltration, and PD-L1 levels, suggesting a tumor-suppressive role via the immune microenvironment [59]. However, in our research, no significant association between *GNA11* expression and survival was observed in patients with stage I–II early RCC. This stage-dependent pattern may be partly attributable to the relatively favorable prognosis and limited outcome variability among localized RCC cases, which could reduce the ability to detect survival differences associated with individual molecular markers. Additionally, another kinase, *PRKCG*, is overexpressed in RCC and linked to immune infiltration and checkpoint molecules, implying a role in immune evasion and metastasis [60]. Beyond RCC, other genes contribute to tumorigenesis through diverse mechanisms. The lipid transporters *SLC27A1* and *SLC27A4* facilitate fatty acid metabolism to promote melanoma and hepatocellular carcinoma, respectively [37,61]. The kinase *PRKCD* regulates breast cancer metastasis [62], while the phosphatase subunit *PPP2R2C* inhibits the mTOR/S6K pathway, exerting tumor-suppressive effects in glioma and prostate cancer [63,64]. Finally, *LCN2* exhibits tissue-specific roles: it ameliorates lung cancer cachexia when expressed at low levels but promotes malignant progression in breast cancer when overexpressed [65,66]. Collectively, these genes form a critical molecular network in tumorigenesis and progression through mechanisms involving metabolic reprogramming (*PFKM*, *PPARGC1A*), lipid transport (*SLC27A*), kinase signaling (*GNA11*, *PRKCG*, *PRKCD*), phosphatase regulation (*PPP2R2C*), and microenvironment modulation (*LCN2*).

This study represents an exploratory bioinformatics investigation and is therefore subject to the inherent limitations of computational analyses. The prognostic model development and multi-cohort validation were based on publicly available multi-omics datasets, and no in vitro or in vivo experiments were performed to directly elucidate the functional or mechanistic roles of the identified genes. Although the SUMOylation-associated prognostic signature demonstrated robust performance across multiple public datasets, all analyses were based on retrospective cohorts. Prospective clinical validation using independent patient populations, standardized molecular assays, and long-term follow-up data will be required before clinical translation. While immune infiltration analysis suggested an association between the SUMOylation-related prognostic signature and tumor immune characteristics, additional immunotherapy-related evaluations, including immune checkpoint profiling, tumor mutation burden analysis, TIDE prediction, and prospective clinical validation, are required to further determine its predictive value for immunotherapy response. Nevertheless, the major strength of this study is the systematic characterization of SUMOylation-associated molecular patterns across distinct RCC subtypes, resulting in the identification of candidate therapeutic targets and the establishment of a cross-subtype prognostic model that may facilitate future experimental validation and mechanistic investigations. Additionally, the KICH subtype analysis was limited by its relatively small sample size (*n* = 17), which reduced statistical power and warrants cautious interpretation. However, these cases were retained to provide a more comprehensive assessment of molecular heterogeneity among the three major RCC subtypes (KICH, KIRC and KIRP). The findings from the KICH cohort should be considered exploratory and hypothesis-generating, providing preliminary insights that may support future studies with larger independent cohorts and subtype-specific clinical investigations.

In the next stage of research, we will focus on the seven selected RCC cell lines. We will use strategies like gene knockout, knockdown, and overexpression to test how the biomarkers affect cell growth, movement, and immune infiltration. At the same time, we will build mouse models using patient-derived tumor tissues (PDX models). These models will help us evaluate whether targeting these genes can control tumor growth and alter the tumor microenvironment. Together, these experiments will help confirm the prognostic value of the biomarkers.

## 5. Conclusions

SUMOylation-associated genes play a pivotal role in the molecular heterogeneity and prognostic landscape of RCC. Through integrated multiomics analysis, we demonstrated their involvement in subtype classification, prognostic model construction, and modulation of the TIME. The developed prognostic model exhibited strong and reproducible predictive performance, underscoring its potential clinical applicability. Furthermore, immune landscape features were significantly associated with risk stratification, suggesting a heightened sensitivity to immunotherapy in high-risk patients. Ultimately, secondary screening identified three RCC cell lines and five candidate genes as prioritized resources for subsequent molecular and functional investigations. Collectively, these findings offer valuable insights into the biology of RCC and support the advancement of precision medicine and personalized immunotherapeutic strategies.

## Figures and Tables

**Table 1:** Univariate Cox regression analysis of TCGA-RCC samples.

Variables	β	S.E	Z	*p*	HR (95%CI)	N
Gene						
*CREB3L1*	0.30	0.10	3.11	0.00	1.35 (1.12–1.62)	758
*GNA11*	−0.19	0.11	−1.73	0.08	0.83 (0.67–1.03)	758
*PFKM*	−0.21	0.09	−2.33	0.02	0.81 (0.68–0.97)	758
*PPARGC1A*	−0.17	0.08	−2.06	0.04	0.85 (0.72–0.99)	758
*PPP2R2C*	0.25	0.08	3.13	0.00	1.29 (1.10–1.51)	758
*PRKCG*	0.26	0.13	1.96	0.05	1.30 (1.00–1.68)	758
Sex						
male					1.00 (Ref.)	516
female	0.13	0.15	0.90	0.37	1.14 (0.85–1.53)	242
Age						
40–60					1.00 (Ref.)	343
>60	0.47	0.15	3.14	0.00	1.60 (1.19–2.14)	387
<40	0.53	0.40	1.34	0.18	1.70 (0.78–3.70)	28
Stage						
II					1.00 (Ref.)	79
I	−0.24	0.29	−0.83	0.41	0.78 (0.44–1.39)	418
IV	1.90	0.28	6.67	0.00	6.69 (3.83–11.69)	89
III	0.92	0.29	3.22	0.00	2.52 (1.44–4.42)	172
Subtype						
KIRP					1.00 (Ref.)	247
KIRC	0.47	0.18	2.62	0.01	1.60 (1.13–2.27)	494
KICH	0.33	0.48	0.68	0.49	1.38 (0.55–3.52)	17

Abbreviations: β, regression coefficient; S.E, standard error; Z, Z score; *p*, *p* value; HR, hazard ratio; CI, confidence interval; Ref., reference group.

**Table 2:** Multivariate Cox regression analysis of TCGA-RCC Samples.

Variables	β	S.E	Z	*p*	HR (95%CI)	N
Riskscore	0.27	0.13	2.08	0.04	1.31 (1.02–1.69)	758
Sex						
female					1.00 (Ref.)	242
male	−0.10	0.15	−0.68	0.50	0.90 (0.67–1.21)	516
Age						
40–60					1.00 (Ref.)	343
>60	0.44	0.15	2.93	0.00	1.56 (1.16–2.10)	387
<40	0.73	0.40	1.80	0.07	2.06 (0.94–4.55)	28
Stage						
I					1.00 (Ref.)	418
III	1.10	0.19	5.85	0.00	3.01 (2.08–4.36)	172
II	0.24	0.29	0.82	0.41	1.27 (0.72–2.26)	79
IV	2.15	0.19	11.38	0.00	8.56 (5.92–12.40)	89
Subtype						
KIRC					1.00 (Ref.)	494
KICH	0.12	0.46	0.26	0.80	1.13 (0.46–2.77)	17
KIRP	−0.11	0.19	−0.58	0.56	0.90 (0.62–1.30)	247

Abbreviations: β, regression coefficient; S.E, standard error; Z, Z score; *p*, *p* value; HR, hazard ratio; CI, confidence interval; Ref., reference group.

## Data Availability

Primary datasets were obtained from the following databases: GeneCards (https://www.genecards.org/Search/Keyword?queryString=SUMOylation&geneCategories=ProteinCoding (accessed on 20 June 2025)). TCGA (https://portal.gdc.cancer.gov (accessed on 25 June 2025)) and UCSC Xena (https://xenabrowser.net/datapages/ (accessed on 25 June 2025)). Datasets: TCGA Kidney Chromophobe (KICH), TCGA Kidney Clear Cell Carcinoma (KIRC), TCGA Kidney Papillary Cell Carcinoma (KIRP) and PCAWG (specimen centric). All data were obtained from the “gene expression RNAseq” section of each database. KEGG (http://www.genome.jp/kegg (accessed on 25 June 2025)), identify the target signaling pathway by name in the KEGG database, and then visualize the data by selecting the “Gene Pathway Network Map (KEGG)” option in the CNS analysis platform (https://cnsknowall.com/#/Home/Contain/BottomContainBar (accessed on 25 June 2025)). HPA (https://www.proteinatlas.org/ (accessed on 25 June 2025)), just search for the target gene by name. The complete analytical pipeline is illustrated in Figure 12.

## Supplementary Materials

The following supporting information can be downloaded at: https://www.mdpi.com/article/10.3390/cimb48080751/s1.

## Abbreviations

The following abbreviations are used in this manuscript:

AMPK	AMP-Activated Protein Kinase
AUC	Area Under Curve
BP	Biological Processes
CC	Cellular Component
C-index	Concordance index
DCA	Decision Curve Analysis
DEGs	Differentially Expressed Genes
FDR	False Discovery Rate
FG vs. BG	Foreground query gene set vs. genome-wide reference Background universe
GO	Gene Ontology
GSVA	Gene Set Variation Analysis
HIF	Hypoxia-Inducible Factor
HPA	Human Protein Atlas
IHC	Immunohistochemical
IL-17	Interleukin-17
K160	Modification at Lysine 160
KEGG	Kyoto Encyclopedia of Genes and Genomes
KICH	Kidney Chromophobe Renal Cell Carcinoma
KIRC	Kidney Renal Clear Cell Carcinoma
KIRP	Kidney Renal Papillary Cell Carcinoma
K-M survival analysis	Kaplan-Meier survival analysis
LASSO	Least Absolute Shrinkage And Selection Operator
log2FC	log2- transformed Fold Change
MF	Molecular Function
nRPX	normalized Relative Protein Expression
nTPM	normalized Transcripts Per Million
OS	Overall Survival
PCA	Principal Component Analysis
PCAWG	Pan-Cancer Analysis of Whole Genomes
PD-L1	Programmed Cell Death Ligand 1
PML-NBs	Promyelocytic Leukemia Nuclear Bodies
PPARs	Peroxisome Proliferators-Activated Receptors
PPI	Protein-Protein Interactions
RCC	Renal Cell Carcinoma
ROC	receiver operating characteristic
SUMOylation	Small Ubiquitin-Like Modifier
TCGA	The Cancer Genome Atlas
TIME	Tumor Immune Microenvironment
UCSC	University of California Santa Cruz
VHL	Von Hippel-Lindau
WHO	World Health Organization
