# Supplementary material for: SUMOylation-Driven Subtype Heterogeneity and Prognostic Biomarkers in Renal Cell Carcinoma
Source: Curr Issues Mol Biol. 2026 Jul 23;48(8):751. doi: 10.3390/cimb48080751 (PMC13511082; doi:10.3390/cimb48080751)
Supplement: Supplementary file 1 [file cimb-48-00751-s001.zip › cimb-4396810-supplementary.pdf]

Supplementary Materials

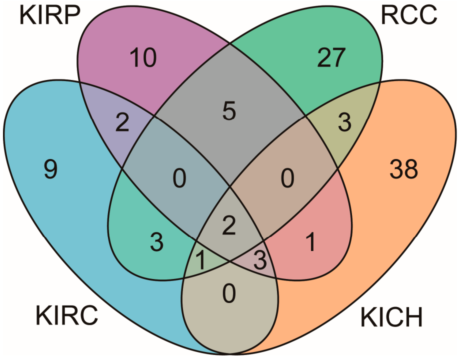

Figure S1. Identification of RCC subtype-specific DEGs.

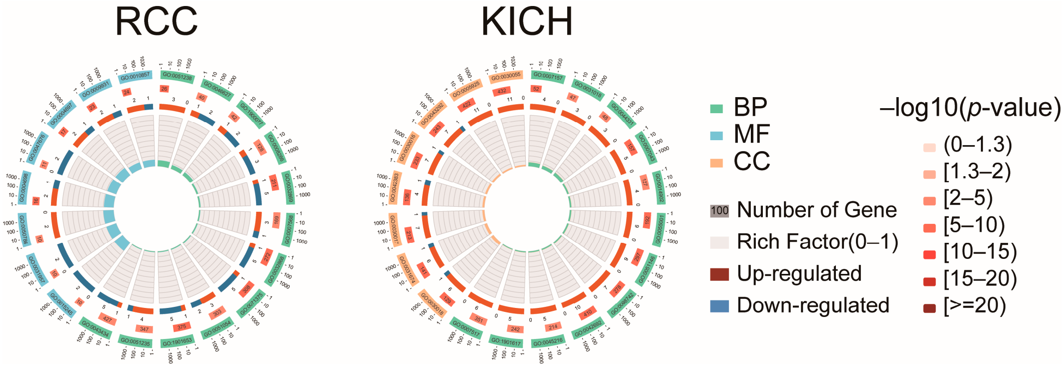

Figure S2. GO enrichment of RCC subtype-specific DEGs. BP, biological process; MF, molecular function; CC, cellular component.

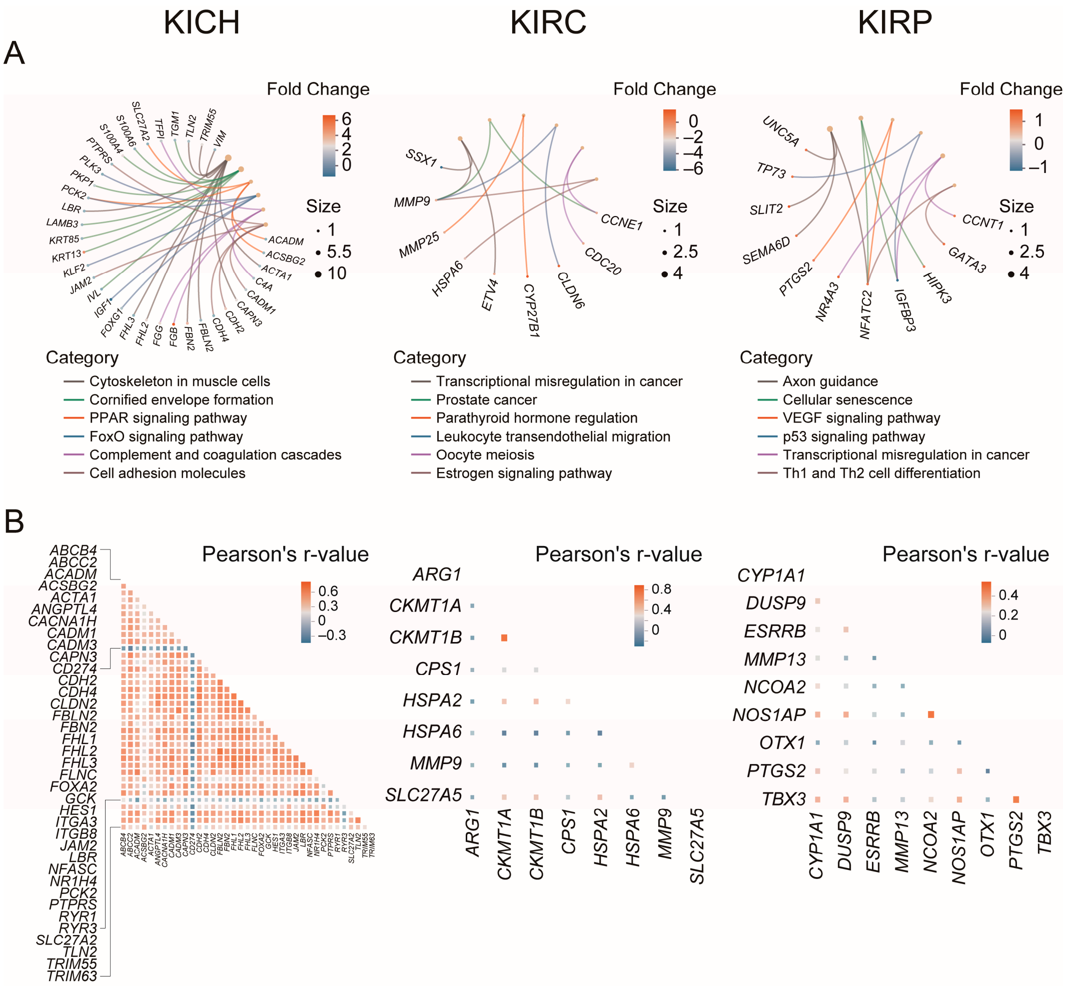

Figure S3. SUMOylation-associated functional analysis of RCC subtypes. (**A**) KEGG pathway enrichment of subtype-specific DEGs. (**B**) Correlation network of subtype-specific DEGs ($p < 0.05$).

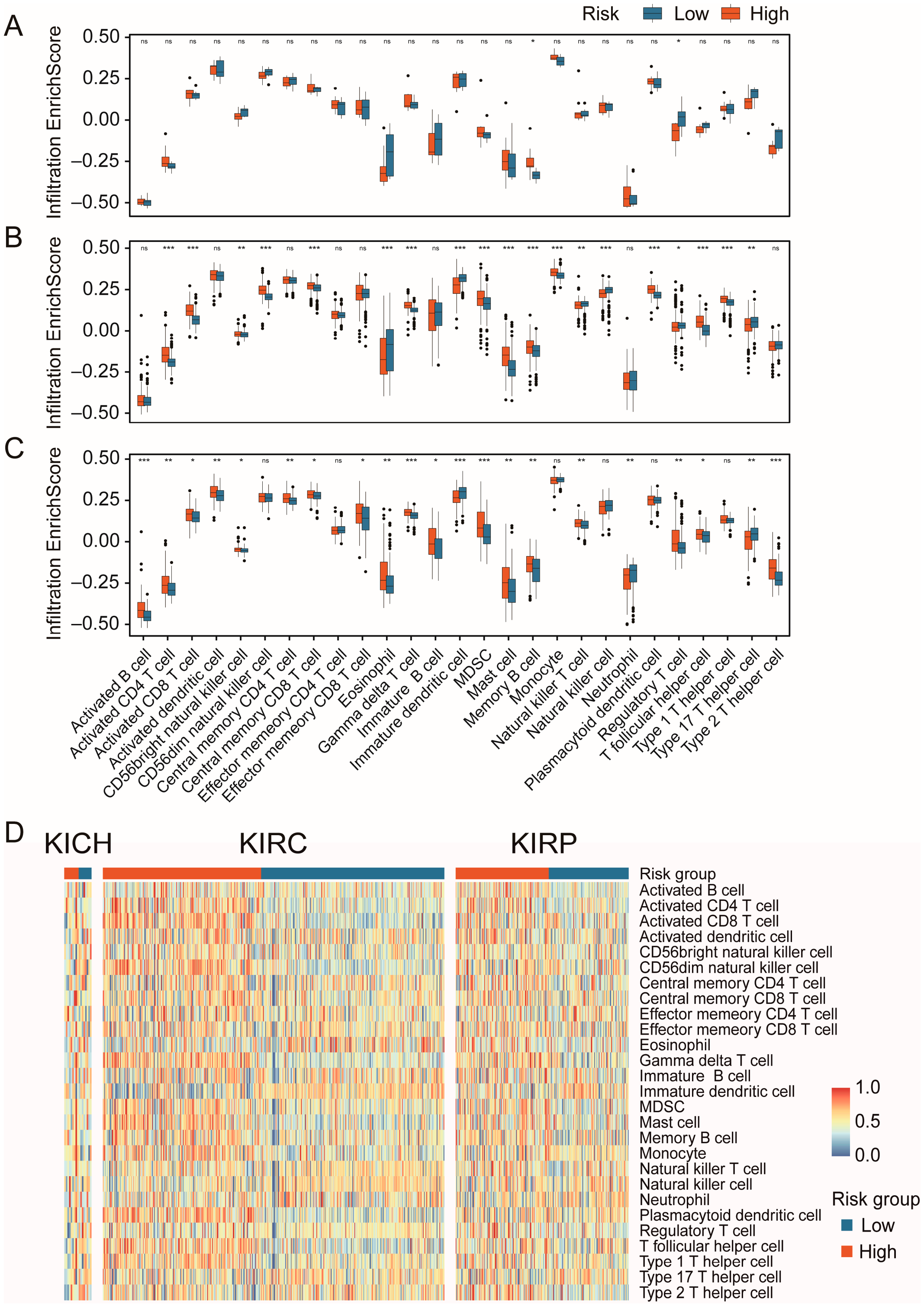

Figure S4. Immune infiltration analysis in the TCGA-RCC cohort. Subtype-specific patterns in KICH (**A**), KIRC (**B**), and KIRP (**C**), and subtype-stratified heatmaps (**D**). ns indicates $p > 0.05$; * indicates $p < 0.05$; ** indicates $p < 0.01$; *** indicates $p < 0.001$.

Table S1. Univariate Cox regression analysis of *GNA11* and clinical features in stage III–IV TCGA-RCC patients.

Variables	β	S.E	Z	P	HR (95%CI)	N
GNA11	-0.86	0.28	-3.03	0.00	0.42 (0.24-0.74)	261
**Sex**						
male					1.00 (Ref.)	179
female	0.10	0.18	0.53	0.60	1.10 (0.77-1.58)	82
**Age**						
>60					1.00 (Ref.)	145
<40	0.23	0.43	0.55	0.59	1.26 (0.55-2.91)	13
40-60	0.05	0.18	0.29	0.77	1.05 (0.74-1.49)	103
**Stage**						
IV					1.00 (Ref.)	89
III	-0.96	0.17	-5.55	0.00	0.38 (0.27-0.54)	172
**Subtype**						
KIRP					1.00 (Ref.)	63
KIRC	0.10	0.22	0.45	0.65	1.11 (0.71-1.71)	191
KICH	0.21	0.54	0.39	0.70	1.23 (0.43-3.55)	7

Abbreviations: β, regression coefficient; S.E, standard error; Z, Z score; P, P value; HR, hazard ratio; CI, confidence interval; Ref., reference group.
